# Identification of protein secretion systems in bacterial genomes

**DOI:** 10.1038/srep23080

**Published:** 2016-03-16

**Authors:** Sophie S. Abby, Jean Cury, Julien Guglielmini, Bertrand Néron, Marie Touchon, Eduardo P. C. Rocha

**Affiliations:** 1 Institut Pasteur, Microbial Evolutionary Genomics, Paris, 75015, France; 2 CNRS, UMR3525, Paris, 75015, France; 3 Institut Pasteur, C3BI, CIB, Paris, 75015, France

## Abstract

Bacteria with two cell membranes (diderms) have evolved complex systems for protein secretion. These systems were extensively studied in some model bacteria, but the characterisation of their diversity has lagged behind due to lack of standard annotation tools. We built online and standalone computational tools to accurately predict protein secretion systems and related appendages in bacteria with LPS-containing outer membranes. They consist of models describing the systems’ components and genetic organization to be used with MacSyFinder to search for T1SS-T6SS, T9SS, flagella, Type IV pili and Tad pili. We identified ~10,000 candidate systems in bacterial genomes, where T1SS and T5SS were by far the most abundant and widespread. All these data are made available in a public database. The recently described T6SS^iii^ and T9SS were restricted to Bacteroidetes, and T6SS^ii^ to *Francisella*. The T2SS, T3SS, and T4SS were frequently encoded in single-copy in one locus, whereas most T1SS were encoded in two loci. The secretion systems of diderm Firmicutes were similar to those found in other diderms. Novel systems may remain to be discovered, since some clades of environmental bacteria lacked all known protein secretion systems. Our models can be fully customized, which should facilitate the identification of novel systems.

Proteins secreted by bacteria are involved in many important tasks such as detoxification, antibiotic resistance, and scavenging^1^. Secreted proteins also have key roles in both intra- and inter-specific antagonistic and mutualistic interactions^2^,^3^. For example, they account for many of the virulence factors of pathogens^4^,^5^. Bacteria with a Lipopolysaccharide-containing outer-membrane (abbreviated “diderm-LPS” in this article) require specific protein secretion systems. Six types of secretion systems, numbered Type I secretion system (T1SS) to Type VI secretion system (T6SS), were well characterised by numerous experimental studies (for some general reviews see^6^,^7^,^8^). The Type IX secretion system (T9SS or PorSS) was more recently uncovered in Bacteroidetes^9^,^10^. In this study, we focused on these diderm-LPS protein secretion systems. A few other systems have been described in diderm-LPS, such as the chaperone-usher pathway, sometimes named Type VII secretion system (T7SS), and the Type VIII secretion system (T8SS). They were not included in this study because they are only involved, respectively, in the export of type I pili and curli^11^. The ESAT-6 secretion system (ESX) system of *Mycobacteria*, named T7SS by some authors^12^, was also excluded from the analysis because it is absent from diderm-LPS bacteria.

The important role of secreted proteins has spurred interest in the production of ontologies and computational methods to categorise^13^ and identify them (Table 1). These are difficult tasks. Firstly, protein secretion systems are large machineries with many different components, some of which are accessory and some interchangeable. Secondly, many of their key components are homologous between systems, which complicates their discrimination. For example, T2SS, T4SS and T6SS include distinct but homologous NTPases^14^. Some bacterial appendages require their own secretion systems to translocate their extracellular components^15^,^16^, and these are sometimes partly homologous to classical secretion systems. For example, several components of the Type IV pilus (T4P) and the Tight adherence (Tad) pilus are homologous to components of the T2SS from *Klebsiella oxytoca*^17^. Thirdly, the sequences of secreted proteins, including extracellular components of the secretion systems, evolve rapidly, thereby complicating the identification of homology by sequence similarity^18^. Fourthly, loci encoding secretion systems are frequently horizontally transferred and lost^19^,^20^, leading to the presence of partial (often inactive) systems in genomes^21^. Finally, experimental studies have focused on a small number of occurrences of each type of system, complicating the assessment of their genetic diversity. On the other hand, secretion systems are often encoded in one or a few neighbouring operons. This information can facilitate the identification of genes encoding secretion systems in genome data^22^,^23^.

Several programs were previously made available to identify components of some, but not all, protein secretion systems (Table 1). These programs are very useful to the biologist interested in browsing the known systems or in annotating a small set of sequences. However, they are web-based, and thus poorly adapted for the analysis of very large datasets. Few of these programs categorise systems as complete or incomplete, and none allows the definition of these parameters. These programs do not identify systems scattered in the chromosome, they only predict components or in some case clusters of components. This limits the detection power, because the ability to re-define the components and genetic organisation of secretion systems facilitates the search for their distant variants^24^.

We have used the vast body of knowledge accumulated from experimental studies of model protein secretion systems to build computational models describing their composition and genetic organization. The models can be plugged in MacSyFinder^25^ to predict protein secretion systems using the standalone application. The pre-defined models can also be used on the webserver version available at http://mobyle.pasteur.fr/cgi-bin/portal.py#forms::txsscan. The results can be visualized with MacSyView^25^. In the standalone application, the users can easily modify the models to change the composition and genetic organisation of the secretion systems. Some of these parameters can also be modified in the webserver version. The accuracy of the models was quantified against an independent dataset of experimentally validated systems. Importantly, we provide models to search for an unparalleled number of protein secretion systems (and some partly homologous systems): T1SS, T2SS (Tad and T4P), T3SS (flagellum), T4SS (conjugation system), T5SS, T6SS^i–iii^, and T9SS. We used the models to search for protein secretion systems in a large panel of bacterial genomes. Previous surveys, mostly dating from a time when few genomes were available, analysed the distribution of some specific protein secretion systems in genomes or metagenomes^17^,^20^,^24^,^26^,^27^,^28^,^29^,^30^,^31^. Due to space limitations, we will not attempt at re-assessing all these works. Instead, we describe our models, show their accuracy, and use them to provide a broad view of the distribution of the different protein secretion systems.

## Results and Discussion

### Overview of the approach

We defined 22 customisable models for the protein secretion systems and related appendages (File S1, Figs 1, 2, 3, 4, 5, 6, Figs S1–S5). This was done in four steps (see Materials and Methods for details): identification of reference and validation datasets, definition of the models, model validation, use of the models to identify the systems in bacterial genomes.

Firstly, we searched the primary literature, reviews and books for references of well-studied systems^9^,^16^,^20^,^26^,^28^,^32^,^33^,^34^,^35^,^36^,^37^,^38^,^39^,^40^. We used them to define two independent datasets (*reference* and *validation*) of experimentally studied secretion systems (Tables S1 and S2).

Secondly, the *reference dataset* was used to define the model and protein profiles for each type of system. The model includes information on the number of components that are *mandatory* (necessarily present in a system), *accessory* (not necessarily present in the system), and *forbidden* (never present in the system). The occurrences of the components were searched using specific hidden Markov model (HMM) protein profiles with HMMER^41^. Protein profiles are more sensitive and specific than Blast-based approaches^42^. Our models used 204 protein profiles (included in the package, File S1), of which 194 were built in our laboratory and the rest taken from public databases (Tables S4 and S5). We decided to build and use our own profiles instead of using those present in public databases, because they showed better specificity and sensitivity for our purpose: predicting and discriminating accurately secretion systems and related appendages. To quantify these trends we searched for profiles with sequence similarity to our profiles in TIGRFAM (the database providing the most specific profiles in our analyses). Only 102 of the 199 profiles not taken from TIGRFAM had significant hits in that database and nearly half of them (48) had multiple hits for the same profile. A table with TIGRFAM profiles matching ours is available (Table S6). The model indicates which genes are co-localised (at less than a given distance relative to contiguous genes in the cluster), and which genes might be encoded elsewhere in the genome (designated *loners*).

Thirdly, the models were validated both in the *reference* and in the independent *validation* (that was not used to design the models and protein profiles) datasets using MacSyFinder (Table 2)^25^. We also compared our results with those of T346Hunter^43^ for T3SS and T6SS (see Supplementary TextS1 and Table S7). We could not make a direct comparison of our results and those of the remaining programs in Table 1 because they do not provide tables with results from the analysis of complete genomes (as T346Hunter does) and they cannot be used locally to analyze large datasets.

Finally, the models were used with MacSyFinder to identify occurrences of each system in 1,528 complete genomes of diderm-LPS species. This procedure retrieved automatically all validly predicted secretion systems (Table S3, and see http://macsydb.web.pasteur.fr). It also retrieved all hits to each component identified in the genomes whether they are part of a protein secretion system or not. In the following sections we describe the models of each type of protein secretion system and the occurrences of the system in bacterial genomes.

### T1SS

**Figure 1:**
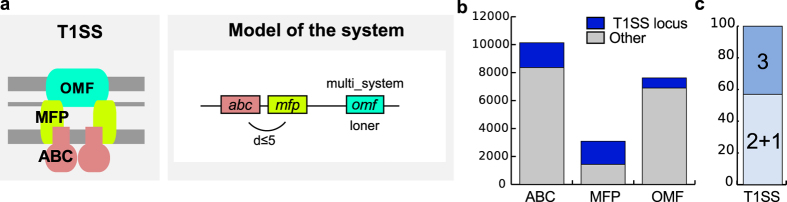
Model and results for the T1SS. (**a**) Schema of the structure (left panel) and model of the genetic organisation (right panel) of T1SS. We built protein profiles for the three components and modelled the two possible genetic architectures of the T1SS: one with the three components encoded in a single locus (*inter_gene_max_space* parameter in MacSyFinder: d ≤ 5 genes), another with the ABC transporter and the MFP encoded in a locus while the OMF is further away (*loner* attribute). A single OMF can also be used by different T1SS^46^ and this is noted by the attribute *multi_system*. (**b**) Distribution of hits for the protein profiles of the T1SS components, separated in two groups: hits effectively part of a T1SS main locus (*i.e.*, containing at least ABC and MFP, blue) and hits found elsewhere (“Other”, grey). Even if encoded outside of “main loci” (grey area of the bar), OMF might be involved in T1SS (*loner* property), whereas it is not the case for ABC and MFP. (**c**) T1SS encoded in one single locus (ABC, MFP and OMF co-localise) (3) or in two (OMF encoded away from the other components) (2 + 1).

We built protein profiles for the three essential components of T1SS^32^,^44^,^45^: the ABC-transporter (ATP-binding cassette transporter) providing an inner membrane channel, the porin (outer membrane factor, OMF) forming the outer-membrane channel, and the inner-membrane anchored adaptor protein (or membrane-fusion protein, MFP) that connects the OMF and the ABC components (Materials and Methods, Tables S4 and S5). T1SS can be difficult to identify because its components have homologs involved in other machineries, *e.g.,* in ABC transporters for the ABC and in drug efflux systems for the MFP, or can themselves be involved in other machineries, in the case of the OMF^46^,^47^,^48^,^49^,^50^. The design of the model was facilitated by the previous observation that genes encoding the ABC and MFP components are always co-localised in T1SS loci^32^,^44^. The model is described in Fig. 1a, and its use resulted in the correct identification of all T1SS in the *reference* and in the *validation* datasets. Overall, 20,847 proteins matched the protein profiles of the T1SS components in the bacterial genomes (Fig. 1b). The vast majority of these were not part of T1SS because they did not fit the T1SS model. We found 1,637 occurrences of the T1SS model in 821 genomes (Fig. 1b). The remaining proteins were probably associated with the numerous other systems carrying components homologous to those of the T1SS.

We found T1SS in more than half of the genomes of diderm bacteria (54%). Some genomes contained many systems; *e.g., Bradyrhizobium oligotrophicum* S58 and *Nostoc sp.* PCC 7524 encoded a record number of 9 systems (Table S3). ABC and MFP were encoded together and OMF apart in more than half (57%) of the T1SS (Fig. 1c). We found 95 loci encoding ABC and MFP in replicons lacking OMF. Many of these systems may be functional, since 94 of these loci were found in genomes encoding at least one OMF in another replicon. Multi-replicon functional T1SS have been previously reported^51^.

### T2SS, T4P and Tad pili

**Figure 2:**
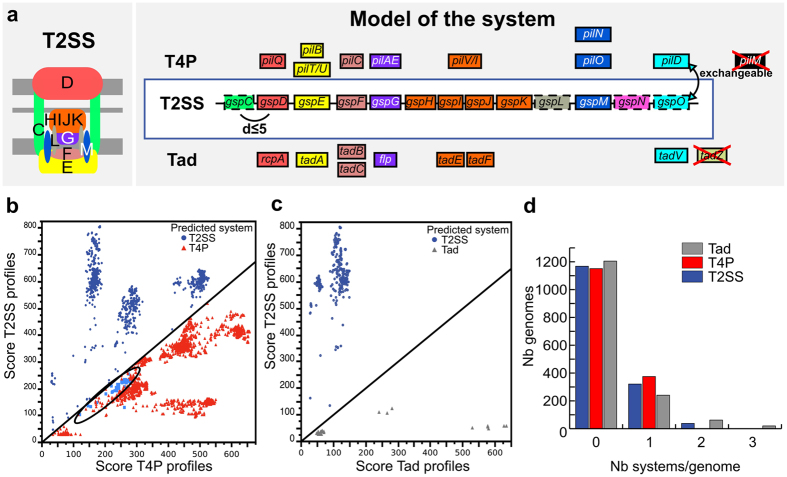
Model of the T2SS for detection and discrimination from the T4 and Tad pili. (**a**) Schema of the structure (left panel) of T2SS, and model of its genetic organisation (right panel), indicating components with homologies with T4P and Tad pilus. We built protein profiles for all these components (Tables S4 and S5). Protein families represented by the same colour are homologous, and their profiles often match proteins from the other systems (except for the Flp and TadE/F families that are less similar). Some prepilin peptidases of T2SS and T4P are defined as functionally interchangeable^109^,^110^,^111^ (curved double-headed arrow, *exchangeable* attribute). Boxes represent components: *mandatory* (plain), *accessory* (dashed) and *forbidden* (red crosses). (**b**) Scores of proteins matched with the profiles of T2SS and T4P. The components of actual T2SS (dark blue) and actual T4P (in red) are well separated, indicating that in each case the best match corresponds to the profile of the correct model system. The exceptions (blue points surrounded by a black ellipse) concern the prepilin peptidases (light blue squares, circled in blue), which are effectively inter-changeable. (**c**) Representation similar to (**b**), but for the comparison between T2SS (blue) and Tad (grey) systems. In this case, the separation is perfect: the proteins always match better the protein profile of the correct system. (**d**) Number of detected systems per genome among the 1,528 genomes of diderm bacteria.

T2SS are encoded by 12 to 16 genes, many of which are homologous to components of the T4P and the Tad pilus^17^,^52^,^53^ (Fig. 2). We used the protein families conserved in the *reference* dataset to build 13 protein profiles for T2SS, 11 for type IV pili, and 10 for Tad pili (Materials and Methods, Tables S4 and S5). We did not build profiles for GspA and GspB because they were rarely identified in T2SS and their alignments were unreliable due to low sequence similarity. The most frequent components in the *reference* dataset were defined as mandatory in the models. The least conserved components were defined as accessory. Some profiles built for one type of system produced matches to (homologous) components of other types of systems. Discrimination between systems was facilitated by the definition of some specific components as forbidden (*e.g.*, GspC was declared forbidden in Tad and T4P).

We identified all of the T2SS and Tad systems of the *reference* dataset using our models for these systems (Table S1). In the *validation* dataset we missed some components of four of the 18 T2SS (Table 2, Table S2), therefore failing to pass the threshold for a complete system in these four cases. For example, we missed the very atypical T2SS of *Legionella pneumophila* because it failed the co-localisation criterion (unusually, it is encoded in five distant loci, Table S2)^54^. The parameters we selected for our default models may be stringent, but MacSyFinder allows to easily modulate them according to the user’s needs. We could for example retrieve three of the four missed T2SS by modifying the default T2SS model, *e.g.*, the “Xps-type” system could be detected by decreasing the required number of components^55^. More relaxed parameters in terms of co-localisation and sequence similarity would have identified all T2SS, but at the cost of less correct discrimination from the two homologous systems, T4P and Tad.

The quality of the default T2SS model was confirmed by the analysis of genomic data. Proteins matched by the protein profiles of T2SS were typically either highly or poorly clustered (Fig. S1a). Clusters with many components were typically part of T2SS, whereas small clusters corresponded to other systems. The T2SS components co-localised much closer than the imposed distance threshold (d ≤ 5, Fig. S1b). The vast majority (99%) of the T4P were encoded in multiple distant loci, which is accepted but not required by the model whereas most T2SS were encoded in one single locus (96.5%). To verify that the T2SS, T4P, and Tad loci were correctly classed, we compared the HMMER scores of proteins matched by protein profiles from different systems. Proteins matching profiles from two types of systems scored systematically higher for the system in which they were classed, *i.e.*, secretins of T2SS were systematically matched with a higher score with the profile for the T2SS (Fig. 2b,c).

We detected 400 T2SS in 360 genomes, 379 T4P in 377 genomes, and 425 Tad pili in 323 genomes. The high abundance of Tad pili is surprising given that they are much less studied than the other systems. Interestingly, we found one Tad pilus with the outer membrane channel (the secretin) in one of the rare Firmicutes with an outer membrane (Clostridia, *Acetohalobium arabaticum* DSM 5501)^56^, and also in Acidobacteria, Chlorobi, and Nitrospirae. T4P, T2SS, and to a lesser extent Tad pili, were usually found in a single copy per genome, but some genomes encoded up to three systems (Fig. 2d). The observed small number of T2SS per genome reinforces previous suggestions that many T2SS might secrete several different proteins^57^.

### T3SS and T4SS

**Figure 3:**
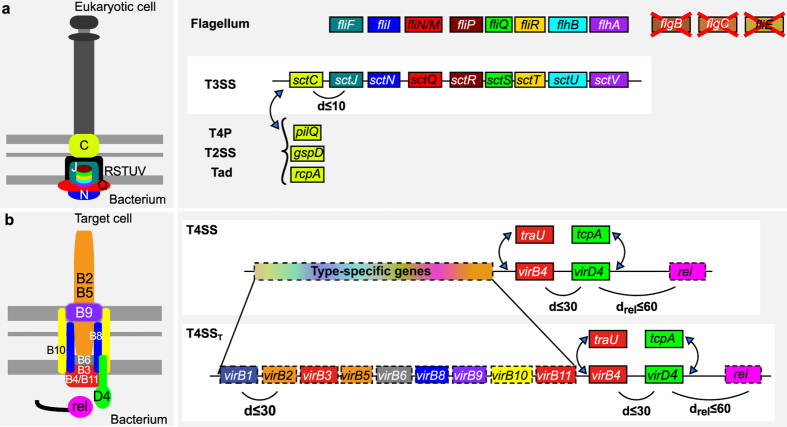
Model of T3SS and T4SS. (**a**) The models of T3SS and flagellum were built based on a previous study^24^ (representation conventions as in Fig. 2). Of the nine *mandatory* components for the T3SS only the secretin is *forbidden* in the model of the flagellum. Conversely, three flagellum-specific components are *forbidden* in the T3SS model. Three different types of secretins are found in T3SS derived from different appendages, which are thus defined as *exchangeable* in the model. (**b**) Models of the T4SS were built based on a previous study^67^. Two different proteins have been described as type 4 coupling proteins (T4CP: VirD4 and TcpA) and two as the major ATPases (VirB4 and TraU, which are homologous). Some pT4SS lack a T4CP and secrete proteins from the periplasm^65^. The relaxase (*rel*), is necessary for conjugation but not for protein secretion, although some relaxase-encoding T4SS are found in both cT4SS and pT4SS^112^,^113^,^114^. Only two MPF types are associated with protein secretion - pT4SS_I_ and pT4SS_T_, corresponding to MPF_I_ and MPF_T_ types. The specificity of type-specific profiles is assessed in Fig. S2.

T3SS and T4SS secrete proteins directly into other cells. The T3SS, sometimes also termed non-flagellar T3SS or NF-T3SS, evolved from the flagellar T3SS (F-T3SS) and is encoded by 15 to 25 genes usually in a single locus^24^,^58^,^59^ (Fig. 3a). Many of the core components of this system are homologous to the distinct F-T3SS that is part of the bacterial flagellum^60^,^61^,^62^. We have previously proposed models that accurately discriminate between the T3SS and the flagellum^24^. We used the same models in this work. We identified 434 NF-T3SS in 334 genomes and 837 flagella in 762 genomes. Some genomes encode many T3SS, *e.g.*, *Burkholderia thailandensis* MSMB121 encodes four T3SS. These results match experimental data showing that in *Burkholderia pseudomallei* the multiple T3SS target different types of cells^63^, and that in *Salmonella enterica* the two T3SS are expressed at different moments in the infection cycle (reviewed in^64^). Multiplicity of T3SS is therefore likely to be associated with complex lifestyles.

T4SS are involved in protein secretion, in conjugation and in some cases in DNA release to, or uptake from, the environment^65^. Here, we distinguished the protein secretion T4SS from the conjugation-related T4SS, which requires a relaxase^66^, by naming them respectively pT4SS and cT4SS. It should be noted that some cT4SS are also able to secrete proteins^65^. We have previously built and validated profiles and models for the pT4SS, and cT4SS^67^ (Fig. 3). The latter can be divided in eight sub-types corresponding to different mating pair formation complexes (MPF)^30^, of which six are found in diderm-LPS bacteria, and only two are known to include pT4SS (MPF_I_ and MPF_T_). To test the specificity of the models of each T4SS sub-type, we studied the close co-occurrence of T4SS components. The results show that most protein profiles are highly specific to each T4SS sub-type (Fig. S2). Hence, our profiles are able to identify and distinguish between these different systems. We identified 156 pT4SS (among 990 T4SS) in 130 genomes of diderm bacteria (Table S3).

### T5SS

**Figure 4:**
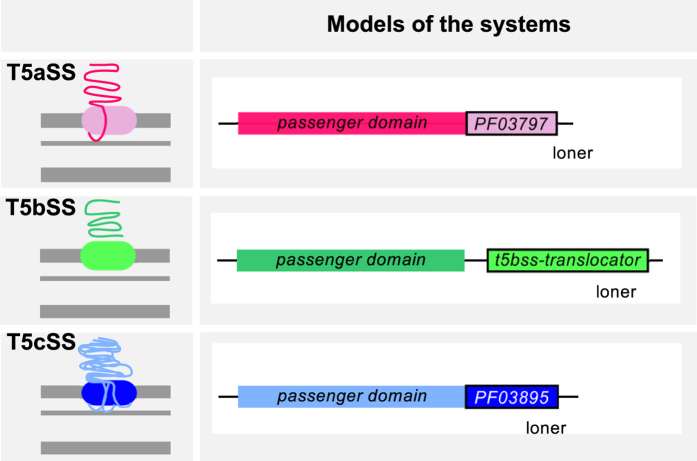
Model of the T5aSS, T5bSS and T5cSS. The left panel shows simplified schemas of the T5SS, and the right panel displays the respective genetic model (only one component that is classed as *loner*). The translocator, pore-forming domains were searched using PFAM domains for T5aSS and T5cSS (resp. PF03797 and PF03895), and a profile built for this work for the T5bSS (Tables S1, S4 and S5).

T5SS are divided in five types (reviewed in^68^,^69^,^70^,^71^). Four types encode the translocator (pore-forming) and the passenger (secreted) domains in a single gene: the classical autotransporter (T5aSS), the trimeric autotransporter (T5cSS), the inverted autotransporter (T5eSS), and the fused two-partner system (T5dSS). In two-partner systems (T5bSS), the translocator and passenger are encoded in two separate (typically contiguous) genes. T5SS rely on the Sec machinery for inner-membrane translocation and require other cellular functions for biogenesis. Many of these functions are ubiquitous in diderm-LPS bacteria and do not facilitate the identification of T5SS. Hence, our models only included information on the conserved, mandatory translocator domain of T5SS (Fig. 4, Fig. S3). Two recently proposed families of T5SS - T5dSS and T5eSS^72^,^73^ - were not matched by the T5SS profiles. We will build specific profiles for the detection of these sub-types when enough experimentally validated examples become available.

Our models were able to identify all T5SS in the *reference* and *validation* datasets, with the exception of an atypical T5bSS of *Pseudomonas aeruginosa* consisting of a translocator domain fused with a component of the chaperone usher pathway^74^. We found 3,829 T5aSS in the genomes of diderm bacteria, which makes them by far the most abundant secretion system in our dataset. Certain *Chlamydiae* genomes contain up to 21 T5aSS. We found 1,125 T5bSS (0–8 per genome) and 849 T5cSS (0–24 per genome). T5SS were encoded in 62% of the genomes of diderm bacteria.

### T6SS

**Figure 5:**
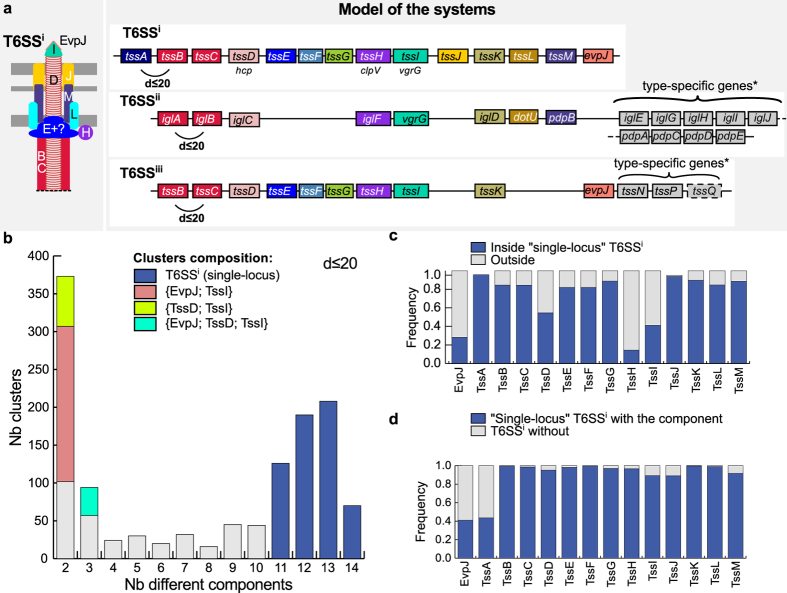
Model and results for the detection of T6SS. (**a**) The left panel shows the schema of the structure of T6SS^i^, and the right panel displays the genetic model of the three sub-types of T6SS (representation conventions as in Fig. 2). For T6SS^i^, we built profiles for the 14 mandatory components, which were clustered if at a distance of d ≤ 20 (see Fig. S4). For T6SS^ii^ and T6SS^iii^, we built 17 and 13 profiles respectively. All components were set as mandatory, except for TssQ, which is found in half of the T6SS^ii^. Homologies between components that are displayed by the mean of the same colours of boxes between the different sub-types are based on previous studies. *Putative type-specific genes are displayed in grey boxes that do not represent homologies. However, several putative homologies were retrieved using Hhsearch (e-value < 1 and p-value < 0.05) on T6SS^ii^ components: iglC (tssG), iglG (tssF), iglH (tssE), iglJ (tssH) and pdpD (tssH). (**b**) Number of different components per cluster of T6SS^i^. Following this analysis, we set the quorum parameter of T6SS^i^ to 11. (**c**) Frequency of hits for each type of T6SS^i^ components in the genomes. Hits matching a single-locus T6SS^i^ are in blue. The other hits match outside the T6SS^i^ loci. (**d**) Frequency of each component within single-locus T6SS^i^. The components EvpJ and TssA were detected in less than 45% of the T6SS^i^, while the other components were found in most T6SS^i^ loci (>89%).

T6SS secrete effectors to bacterial or eukaryotic cells. They were recently divided in three sub-types^40^, among which T6SS^i^ is by far the most studied^75^,^76^,^77^,^78^,^79^,^80^,^81^. This sub-type has more than a dozen components^78^,^82^. We built profiles for 14 conserved protein families (Fig. 5a, Materials and Methods, Tables S1, S4 and S5), of which 13 were previously described as the most conserved components of the T6SS^i^ 20. The remaining profile corresponds to the PAAR-repeat-containing EvpJ protein family of the spike complex^83^, present in eight out of the nine T6SS^i^ in the *reference* dataset. Using this model we identified all T6SS^i^ of the *reference* and *validation* datasets. We only found an inaccuracy in *Escherichia coli* O42 where two systems adjacent in the genome were identified as a single system. Part of the T6SS^i^ machinery is structurally homologous to the puncturing device of phages, from which it may have originated^84^. Yet, our model did not identify a T6SS^i^ in any of the 998 phages present in GenBank, showing that it does not mistake puncturing devices for components of the T6SS.

We identified 652 T6SS^i^ in 409 bacterial genomes, with up to six T6SS^i^ per genome in some *Burkholderia pseudomallei* strains. Around 9% of the T6SS^i^ were encoded in multiple loci in the genome. Interestingly, 35% of the replicons encoding a T6SS^i^ encoded TssI (VgrG) away from the main loci, with a PAAR-containing component (EvpJ) and/or the chaperone TssD (Hcp) (Fig. 5b–d). PAAR-motifs promote the physical interaction between VgrG and toxins, which are often encoded in the same locus^81^,^83^,^85^. It has recently been proposed that VgrG might also be involved in toxin export in a T6SS-independent way^81^. Genomes lacking T6SS^i^ did carry some of these small *tssI*-associated clusters, although this corresponded to only 8% of the clusters. Hence, the study of the loci encoding TssI might uncover new T6SS^i^ effectors.

The T6SS^ii^ sub-type described in *Francisella tularensis*, is involved in the subversion of the immune system (growth in macrophages)^39^,^86^,^87^,^88^. Three of the components of the T6SS^ii^ were seldom matched by T6SS^i^ profiles (*tssBCL*), complicating the detection of T6SS^ii^ with the T6SS^i^ model. We built 17 protein profiles and made a specific model for T6SS^ii^ based on a *Francisella tularensis* system (see Fig. 5, Materials and Methods and Table S5)^88^,^89^. Using HHsearch^90^, we confirmed the existence of weak sequence similarity between the proteins encoded by *tssBCIL* and T6SS^i^ and/or T6SS^iii^ components (p-value < 0.001). The model detected 30 T6SS^ii^ in bacterial genomes. All instances were identified exclusively within the 18 genomes of *Francisella*, and all genomes of the genus contained at least one system.

A recent report identified a new type of T6SS^iii^ involved in bacterial competition in *Flavobacterium johnsoniae*^40^. This sub-type lacked homologs of the “trans-envelope subcomplex” and included nine homologs of the 13 described core components of T6SS^i^ (Fig. 5). Furthermore, it had three specific components (TssN, TssO and TssP) that are absent in the other sub-types of T6SS. Only three loci were reported for this sub-type^40^. We used them to build 13 protein profiles, including the 12 abovementioned proteins and EvpJ. We could not build a protein profile for TssO because of the lack of sufficient representative conserved sequences (Table S5). The parameters of the model were inferred from the analysis of the clusters of hits for T6SS^iii^ components’ profiles (Fig. S4), and from the three reference loci. We predicted 20 T6SS^iii^ in 18 of the 97 Bacteroidetes genomes. TssQ, which was not previously recognized as conserved, was found in 50% of the systems’ occurrences. The family of TssQ proteins matched no PFAM profile, but using InterProScan we could predict the presence of one secretion signal and its cellular localisation at the outer-membrane^91^,^92^. Interestingly, we could find occurrences of EvpJ (harbouring a PAAR domain) within 6 of the 20 T6SS^iii^ main loci, and outside of the main locus in 6 genomes with a T6SS^iii^. This component co-localised with TssD (Hcp), TssE, or TssD and TssI (VgrG). This suggests it might have similar roles in T6SS^i^ and in T6SS^iii^. T6SS^iii^ was only identified among Bacteroidetes.

### T9SS

**Figure 6:**
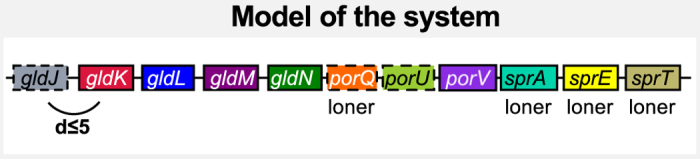
Genetic model of the T9SS. The representation follows the conventions of Fig. 2. The model includes 11 components for which 13 protein profiles were obtained from PFAM (SprA and SprA-2), TIGRFAM (GldJ, GldK, GldL, GldM, GldN and SprA-3) or designed for this study (PorU, PorV, PorQ, SprE, SprT). Four components were declared as *loners*. The co-localisation distance for the others was set at d ≤ 5 (see Fig. S5). As several profiles were available for SprA, we included them all in the models, and declared them as exchangeable homologs in the model. GldJ is not part of the secretion system, but of the gliding motility system. It was included in the model as it facilitates the detection of T9SS components that co-localise with it.

A novel protein secretion system, T9SS or PorSS, has been described in *F. johnsoniae* and *Porphyromonas gingivalis*^9^,^93^. It is required for the secretion of components of the gliding motility apparatus, adhesins and various hydrolytic enzymes. We used eight protein profiles from TIGRFAM and PFAM for five components (some having several profiles), and built protein profiles for five other components (Tables S4 and S5, Materials and Methods). One of the profiles was not specifically associated with T9SS; it is part of the gliding motility machinery (GldJ). It was included in the model because it clusters with some of the T9SS components and thus facilitates their identification. Hence, our model for the T9SS includes 13 protein profiles for 10 core components^93^ (Fig. 6, Fig. S5). To reflect the reference systems’ genetic organization, four components of the T9SS were defined as scattered (*loners*) in the chromosome, whereas the others were defined as part of gene clusters (default behaviour). We detected 60 T9SS in 60 of the 97 genomes of Bacteroidetes, and none in the other bacterial clades, as previously shown^10^. T9SS were found in 62% of the species of Bacteroidetes.

### Distribution of secretion systems

**Figure 7:**
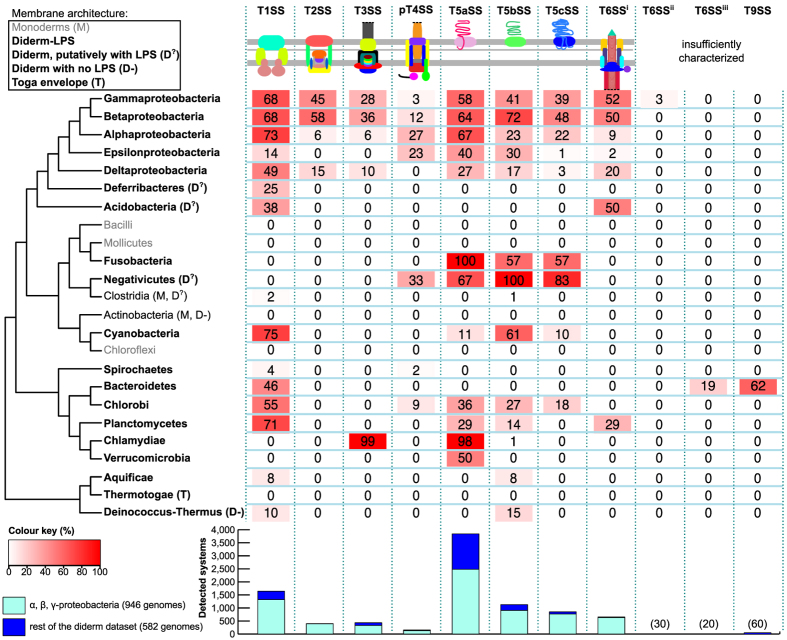
Phylogenetic distribution of protein secretion systems in bacteria. Within each clade, the proportion of genomes harbouring each system is indicated in boxes whose colours follow a gradient from full red (100%) to white (0%) (see legend). Clades were classed as monoderms (grey or “M” symbol), diderms with Lipopolysaccharide-containing outer membranes (Diderm-LPS in bold, no symbol), diderms with homologs of LPS pathway that putatively have LPS (D^?^) and diderms with no LPS (D−). The peculiar envelope of the Thermotogae is indicated (T). The Firmicutes are typically monoderms, but some of their members are diderms (the Negativicutes, some Clostridia, Mycobacteria). The bar plot shows the number of detected systems. Bars are split in two categories to separate on one side Alpha- Beta- and Gamma-proteobacteria, and on the other genomes from other bacteria. We display the number of occurrences of systems occurring rarely in our dataset on top of the bars. Clades with less than 4 genomes and/or with unreported phylogenetic position are not shown (*i.e.*, Chrysiogenetes, Gemmatimonadetes, Nitrospirae and Thermodesulfobacteria). This sketch tree was drawn from the compilation of different published phylogenetic analyses^115^,^116^,^117^,^118^.

To the best of our knowledge, this is the first report comparing the frequency of all well-known protein secretion systems of diderm-LPS bacteria in bacterial genomes. Therefore, we analysed the distribution of these protein secretion systems in relation to bacterial phylogeny, including clades with more than four genomes and with reliable information on their phylogenetic position (Fig. 7). Only three clades, Alpha-, Beta- and Gamma-Proteobacteria, encoded all the six most-studied protein secretion systems (T1-T6SS^i^). Delta- and Epsilon-Proteobacteria showed fewer or no T2SS, T3SS and pT4SS. Most other clades encoded fewer types of systems. The distributions of T3SS, T4SS, T6SS, and T9SS have been described recently^10^,^20^,^24^,^30^,^40^, so we shall focus our analysis on the other systems.

T1SS and T5SS are the most widespread protein secretion systems (Fig. 7, see below). We predicted T1SS in phyla as diverse as Spirochaetes, Planctomycetes, Aquificae, Bacteroidetes, and Cyanobacteria. T1SS involved in the secretion of glycolipids for heterocysts formation were recently described in filamentous Cyanobacteria^94^,^95^. We found that T1SS were particularly abundant in this clade as 75% of the genomes harboured at least one T1SS. Overall, the three types of T5SS showed similar taxonomic distributions, even if T5cSS were less widespread (Fig. 7). Some phyla had only one type of T5SS: T5aSS in Thermodesulfobacteria and T5bSS in Aquificae and Deinococcus-Thermus. There were few genomes available for these clades. Further work will be needed to know if they lack the other T5SS.

We predicted very few T2SS outside Proteobacteria. T2SS were also absent from the 98 genomes of Epsilon-proteobacteria. We found a T2SS in a non-Proteobacterium, *Desulfurispirillum indicum* S5, a free-living spiral-shaped aquatic Chrysiogenetes (also encoding a T1SS). We could not find a description of the membrane architecture for this species, but our analysis reinforces previous suggestions that it is a diderm^96^. Putative T2SS were previously identified in clades where we failed to identify complete systems: *Synechococcus elongatus* (Cyanobacteria), *Chlamydia trachomatis* (Chlamydiae) and *Leptospira interrogans* (Spirochaetes)^28^,^97^,^98^,^99^. The cyanobacterial system, which has a role in protein secretion and biofilm formation, seems to be a typical T4P encoded in multiple loci. The role of T4P in secreting proteins that are not part of its structure has been described before^100^. To the best of our knowledge, the function of the *Leptospira* system was not experimentally tested. The Chlamydiae system was indeed associated with protein secretion^98^. From the point of view of our models the putative T2SS from these two last clades form incomplete systems (although they could be retrieved by lowering the minimum required number of components for a valid T2SS in the model). Preliminary phylogenetic analyses did not allow conclusive assignment of these systems to T2SS or to T4P. Further experimental and computational work will be necessary for their precise characterisation.

### Secretion systems and the cell envelope

The distribution of secretion systems is linked with the structure of the cell envelope. Expectedly, all genomes of monoderms lacked loci encoding diderm-like protein secretion system. Several clades of diderm bacteria lacked many types of protein secretion systems, but only one lacked them all: the Thermotogae. These bacteria are thermophilic, and one could speculate that high temperatures could be incompatible with the protein secretion systems that we searched for. Yet, life under high temperatures is also typical of the sister-clade Aquificae, where we found T1SS and T5SS. The lack of typical protein secretion systems in Thermotogae might be caused by the peculiar sheath-like structure present in their outer cell envelope, the “toga”^101^. This may have led to the evolution of secretion systems specifically adapted to this structure. Accordingly, only a few porins have been identified so far in Thermotogae^102^. In an analogous way, *Mycobacteria* (Actinobacteria), which have a peculiar mycolate outer membrane, have specific secretion systems^12^.

The cell envelope of recipient cells is also a key determinant of the evolution of systems secreting effectors directly into other cells. The extracellular structures of T3SS are tightly linked with the type of eukaryote cell (plant vs. animal) with which the bacterium interacts^24^.

Interestingly, diderm bacteria in taxa dominated by monoderms have protein secretion systems homologous to those of Proteobacteria (including Clostridia, Cyanobacteria, Fusobacteria and Negativicutes). For example, we predicted in Negativicutes (a clade of Firmicutes) putative pT4SS and the three types of T5SS. Some genomes of Halanaerobiales (a sub-clade of Clostridia, Firmicutes) encode T1SS and T5bSS. Similarities in the cell envelope may thus lead to the presence of similar systems in very distant bacteria.

## Conclusion

We were able to identify nearly all protein secretion systems in both the *reference* and the *validation* datasets. The few missed systems were either very atypical (such as the scattered T2SS of *Legionella*) or included components very divergent in sequence (several T2SS). In the latter case, the relaxation of the parameters of the T2SS model allowed their identification. We emphasize that our models are publicly available and can be modified by the user to increase their sensitivity. Relaxing the parameters for the detection of the components (HMMER i-evalue and profile coverage), or for the genetic organization (required quorum of components, co-localisation criterion) often allowed retrieving more putative systems. We emphasize that we have not modified the default models in function of the validation procedure because that would have made our validation procedure inaccurate. Yet, the user is free to take the default models and make them less strict. Nevertheless, this might lead to an increased number of false positives. Complementary analyses can also facilitate the identification of systems. For example, when multiple profiles match a given protein, the one of the system usually provides the highest score (Fig. 2b,c). This is one of the advantages of using specifically designed protein profiles, instead of generic profiles as can be found in PFAM: the system-specific profiles distinguish between homologs components in different types of molecular systems.

We may have under-estimated the presence of protein secretion systems in poorly sampled phyla because of the rapid evolution of extracellular components and the paucity of experimental data. Yet, several pieces of evidence suggest that we may have identified most systems. 1) We identified almost all known systems in the *reference* and *validation* datasets. 2) We identified at least one type of secretion system in almost all clades of diderm bacteria. 3) We identified components of T4P and Tad (homologous to T2SS), F-T3SS (homologous to NF-T3SS), and cT4SS (homologous to pT4SS) with profiles for the protein secretion systems in many clades, including monoderms (Table S3). Most of these systems are monophyletic. If our protein profiles match homologs in outgroup systems, then they probably match all occurrences of the system. Given these arguments, it is tempting to speculate that currently unknown protein secretion systems remain to be discovered in clades where few or no secretion systems could be identified. Interestingly, the recently discovered T6SS^iii^ and T9SS are restricted to Bacteroidetes^9^,^40^, while T6SS^ii^ are only found in *Francisella*. The search for protein secretion systems and other cellular appendages with relaxed criteria may help in identifying novel unknown systems.

## Materials and Methods

### Data

The genomes of bacteria (2,484) and archaea (159) were downloaded from NCBI RefSeq (ftp://ftp.ncbi.nih.gov/genomes/, November 2013). We took from this dataset the 1,528 genomes of bacteria classed as diderm in the literature^103^,^104^,^105^ (Table S3). A total of 998 genomes of phages were downloaded from Genbank (last access, February 2013). The sequences of the reference protein secretion systems were retrieved from Genbank or from complete genomes (Tables S1 and S2).

### Systems definition and identification

We built a dataset of experimentally studied secretion systems (T1SS-T6SS, T9SS) and related appendages (Tad, T4P and the bacterial flagellum) from the analysis of published data. We selected these systems in order to maximise sequence diversity. They form our *reference* set of systems (Table S1). This *reference* dataset was used to build the models and the corresponding HMM protein profiles (see below) of each system using MacSyFinder. This software is publicly available^25^. A detailed explanation of this program can be found in its original publication^25^. Here, we focus on the features that are pertinent for this work. A model in MacSyFinder defines the components of the secretion system, the minimal acceptable number of components, and their genetic organisation. Among other things (see http://macsyfinder.readthedocs.org for full documentation), one can specify the following relevant information. 1) Systems are encoded in a single locus (*single-locus* system) or in several loci (*multi-loci* system). 2) Core components (ubiquitous and essential) are defined as *mandatory*. 3) Components that are accessory or poorly conserved in sequence are defined as *accessory*. These components are accessory for the computational model, but their function may be essential. This happens when different proteins have analogous functions or when proteins evolve so fast that distant homologs are not recognisable by sequence analysis. 4) Some genes are ubiquitous and specific to a system and can be defined as *forbidden* in models of other systems. This facilitates the discrimination between systems with homologous components. For example, the NF-T3SS-specific secretin may be declared as *forbidden* in the F-T3SS. 5) An occurrence of a system is validated when a pre-defined number (*quorum*) of mandatory components and/or sum of mandatory and accessory components is found^25^. 6) Components can be defined as reciprocally *exchangeable* in the quorum (which prevents them from being counted twice). 7) Two components are co-localised when they are separated by less than a given number of genes (parameter *d* = *inter_gene_max_space*). 8) A component defined with the *loner* attribute does not need to be co-localised with other components to be part of a system (*e.g*., OMF in T1SS). 9) A component that can participate in several instances of a system (*e.g.,* OMF in T1SS) receives the *multi_system* attribute. These different properties can be combined when necessary.

The models for the different protein secretion systems were described using a dedicated Extensible Markup Language (XML) grammar^25^. The files with the models were named after the system (*e.g.,* T1SS.xml, File S1). Models can be easily modified on the standalone version of MacSyFinder. The webserver allows the use of the pre-defined models and the modification of the most important search parameters.

MacSyFinder was used to identify protein secretion systems in bacterial genomes in three steps (for corresponding command-lines see the README file in File S1, and for a full description of the software see^25^). Firstly, components were identified using protein profile searches with HMMER^41^. Hits with alignments covering more than 50% of the protein profile and with an i-evalue < 10^−3^ were kept for further analysis (default parameters). Secondly, the components were clustered according to their proximity in the genome using the parameter *d*. Finally, the clusters were validated if they passed the criteria specified in the model.

### Definition of protein profiles

The models include 204 protein profiles. The two profiles for T5aSS and T5cSS were extracted from PFAM^68^,^91^. Eight profiles for T9SS were extracted from PFAM or TIGRFAM^91^,^106^. The remaining 194 profiles were the result of our previous work^24^,^67^,^107^ or this study (84 protein profiles for T1SS, T2SS, Tad, type IV pilus, T5bSS, T6SS^i^, T6SS^ii^, T6SS^iii^ and T9SS, listed in Table S4). To build the new profiles, we sampled the experimentally studied systems from our *reference* set of systems for proteins representative of each component of each system. Protein families were constructed by clustering homologous proteins using sequence similarity. The details of the methods and parameters used to build each protein profile are described in Table S5. In the case of the T9SS, where only two systems were experimentally characterised, we used components from the well-studied system of *F. johnsoniae* (or *P. gingivalis* when the gene was absent from *F. jonhsoniae*) for Blastp searches against our database of complete genomes, and retained the best sequence hits (e-value < 10^−20^) to constitute protein families. A similar approach was taken to build protein profiles for the T6SS^ii^, based on the *Francisella tularensis subsp. tularensis* SCHU S4 FPI system displayed in Table 1 of^39^. The largest families were aligned and manually curated to produce hidden Markov model profiles with HMMER 3.0^41^.

### Availability

Detection and visualization of all systems described in this paper can be performed online on the Mobyle-based^108^ webserver TXSScan: http://mobyle.pasteur.fr/cgi-bin/portal.py#forms::txsscan. Detection can also be performed locally using the standalone program MacSyFinder^25^, and the sets of models and profiles described here. MacSyFinder is freely available for all platforms at https://github.com/gem-pasteur/macsyfinder. Models and required protein profiles are available as supplemental material (File S1) at https://research.pasteur.fr/en/tool/txsscan-models-and-profiles-for-protein-secretion-systems. The models are provided as simple text (XML) files, so they can be easily modified and extended by the user. The results of MacSyFinder can be visualized with MacSyView, available online at http://macsyview.web.pasteur.fr or for download at https://github.com/gem-pasteur/macsyview (also included in the release of MacSyFinder). The systems detected in this study are available on the form of a database at http://macsydb.web.pasteur.fr.

## Additional Information

**How to cite this article**: Abby, S. S. *et al.* Identification of protein secretion systems in bacterial genomes. *Sci. Rep.* **6**, 23080; doi: 10.1038/srep23080 (2016).

## Supplementary Material

Supplementary Information

Supplementary Table S3

Supplementary Table S7

## Figures and Tables

**Table 1:** Public webservers (Web) and downloadable applications (App) to identify components (C), clusters of components (CC), or complete (eventually scattered, S) bacterial protein secretion systems.

Name	System	Web	App	C (method)	CC	S	URL and Reference
AtlasT4SS	T4	Yes	No	Yes (Blast)	No	No	http://www.t4ss.lncc.br^119^
CONJscan	T4	Yes	No	Yes (HMM)	No	No	http://mobyle.pasteur.fr/cgi-bin/portal.py#forms::CONJscan-T4SSscan^67^
SecReT4	T4	Yes	No	Yes (Blast or HMM)	Yes	No	http://db-mml.sjtu.edu.cn/SecReT4^120^
SecReT6	T6^i–ii^	Yes	No	Yes (Blast or HMM)	Yes	No	http://db-mml.sjtu.edu.cn/SecReT6^121^
SSPred	T1, T2, T3, T4	Yes	No	Yes (other: amino acid composition)	No	No	http//www.bioinformatics.org/sspred^122^
T346Hunter	T3, T4, T6^i^	Yes	No	Yes (Blast and HMM)	Yes	No	http://bacterial-virulence-factors.cbgp.upm.es/T346Hunter^43^
T3DB	T3	Yes	No	Yes (Blast)	No	No	http://biocomputer.bio.cuhk.edu.hk/T3DB/browse^123^
T3SSscan	T3	Yes	No	Yes (HMM)	No	No	http://mobyle.pasteur.fr/cgi-bin/portal.py#forms::T3SSscan-FLAGscan^24^
TXSScan	T1, T2, T3, T4, T5 (a, b, c), T6^i–iii^, T9, T4P, Tad, flagellum.	Yes	Yes	Yes (HMM)	Yes	Yes	http://mobyle.pasteur.fr/cgi-bin/portal.py#forms::txsscan This work

**Table 2:** Summary of experimentally validated systems detected by TXSScan.

Model	Systems detected in the *validation*dataset* (detected/total)	Systems detected in the *reference* dataset (detected/total)
T1SS	6/6	8/8
T2SS	14/18	9/9
T3SS	See^24^	See^24^
T4SS	See^67^,^107^	See^67^,^107^
T5aSS	4/4	NA (PFAM)
T5bSS	2/3	6/6
T5cSS	2/2	NA (PFAM)
T6SS^i^	8/8#	9/9
T6SS^ii^	NA	1/1
T6SS^iii^	NA	3/3
T9SS	NA	2/2

^*^The *validation* dataset was used to test the validity of the models and profiles built from the *reference* dataset.

^#^Two contiguous T6SS were predicted as a single system (see main text) in *Escherichia coli* O42.
