# Supplementary material for: Identification of protein secretion systems in bacterial genomes
Source: Sci Rep. 2016 Mar 16;6:23080. doi: 10.1038/srep23080 (PMC4793230; doi:10.1038/srep23080)
Supplement: Supplementary Information [file srep23080-s1.pdf]

Supplemental Information for:

Identification of protein secretion systems in bacterial genomes

Sophie S Abby [a,b,#,*], Jean Cury [a,b], Julien Guglielmini [a,b,+], Bertrand Néron [c], Marie Touchon [a,b], Eduardo PC Rocha [a,b]

[a] Institut Pasteur, Microbial Evolutionary Genomics, Paris, 75015, France

[b] CNRS, UMR3525, Paris, 75015, France

[c] Institut Pasteur, C3BI, CIB, Paris, 75015, France

*Address correspondence to sophie.abby.univ@gmail.com

Present addresses:

[#] Division of Archaea Biology and Ecogenomics, Department of Ecogenomics and Systems Biology, University of Vienna, A-1090 Vienna, Austria

+ Bioinformatics and Biostatistics HUB, Center of Bioinformatics, Biostatistics and Integrative Biology (C3BI), Institut Pasteur, Paris, 75015, France

Table of contents

Figure S1. Analyses to define the parameters for T2SS detection. 3
Figure S2. Co-occurrence matrix of the MPF-sub-types components for diderms. 4
Figure S3. Analyses to define the parameters for T5SS detection. 6
Figure S4. Analyses to define the parameters for T6SS^{i} and T6SS^{iii} detection. 8
Figure S5. Analyses to define the parameters for T9SS detection. 10
Table S1. Reference dataset: experimentally validated systems used to build HMM protein profiles for bacterial secretion systems and related appendages. 11
Table S2. Validation dataset: detection of experimentally validated systems. 13
Table S3. Distribution of protein secretion systems in the analysed dataset. 17
Table S4. List of the HMM protein profiles included in the study. 18
Table S5. Methods employed to build HMM protein profiles. 23
Table S6. TIGRFAM profiles matching the TXSScan profiles. 24
Table S7. Comparison of the number of predicted T3SS and T6SS^{i} between TXSScan and T346Hunter. 31
Text S1. Comparison of TXSScan predictions of T3SS and T6SS with T346Hunter's predictions 31
File S1. Models and profiles for the detection of protein secretion systems and related appendages with MacSyFinder. 34
References 35

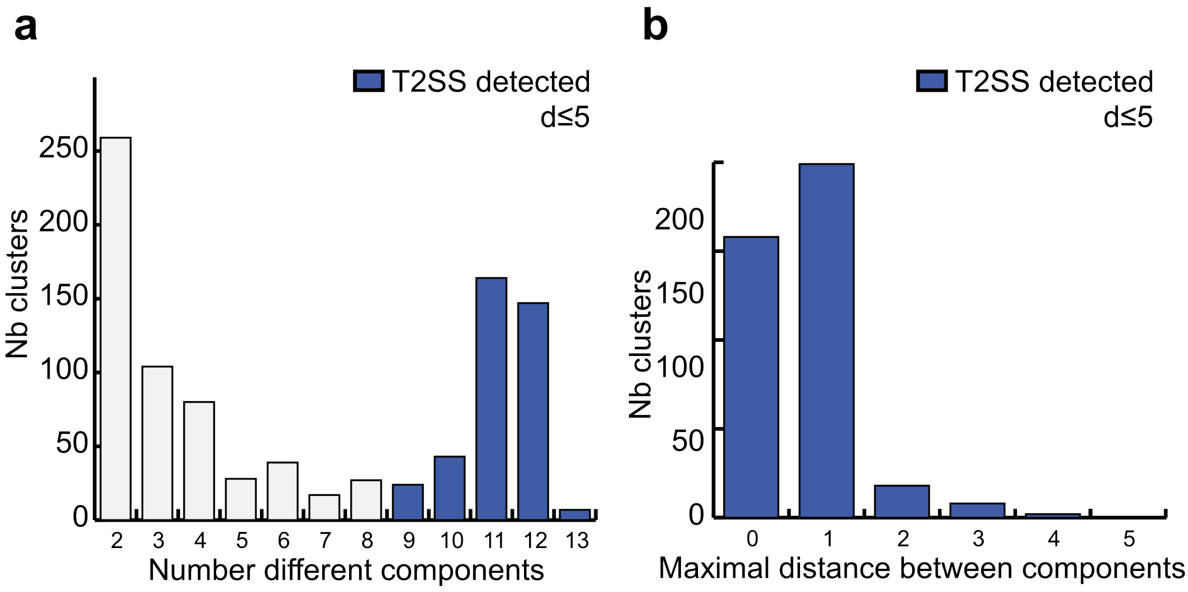

Figure S1. Analyses to define the parameters for T2SS detection.

a. Distribution of the number of different homologs of components of T2SS per cluster using the co-localization parameter d≤5. **b.** Distribution of the maximal distance between consecutive components of T2SS clusters. Given the distributions in **a** and **b**, the minimal number of components required to validate a T2SS (dark blue in the figure) was set to 9 (of which 6 for mandatory components). The parameter d≤5 corresponds to the tail of the observed maximal distance in panel **b**.

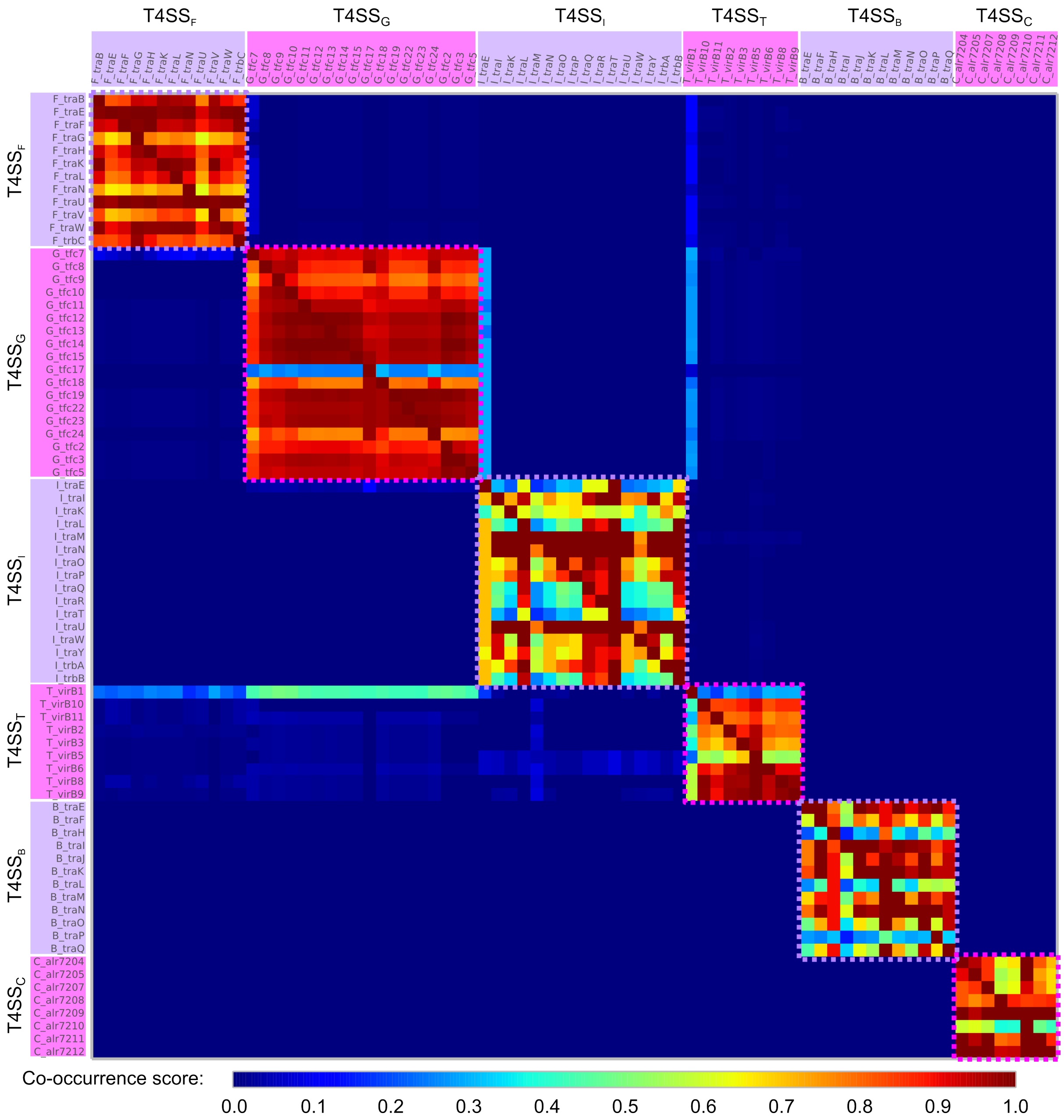

Figure S2. Co-occurrence matrix of the MPF-sub-types components for diderms.

For each pair of hits, the proportion of their co-occurrence in a cluster (as defined in Figure 3) is displayed on a heatmap using a gradient of colours. In the lower-left triangle, this proportion is normalized by the total number of hits of the component in the row, and resp., in the upper-right triangle, it is normalized by the total number of hits of the component in the column. This graph illustrates the specificity of the profiles, since a profile specific of one sub-type of T4SS (MPF type in subscript) is almost never found co-occurring with a profile specific of another sub-type of T4SS. The horizontal and vertical lines showing less specificity concern VirB1, the transglycosylase, which has homologs in other T4SS types that cross-match in terms

of protein profiles. The MPF types found in diderm genomes are MPF_C, MPF_G, MPF_F, MPF_I, MPF_T, and MPF_B [1].

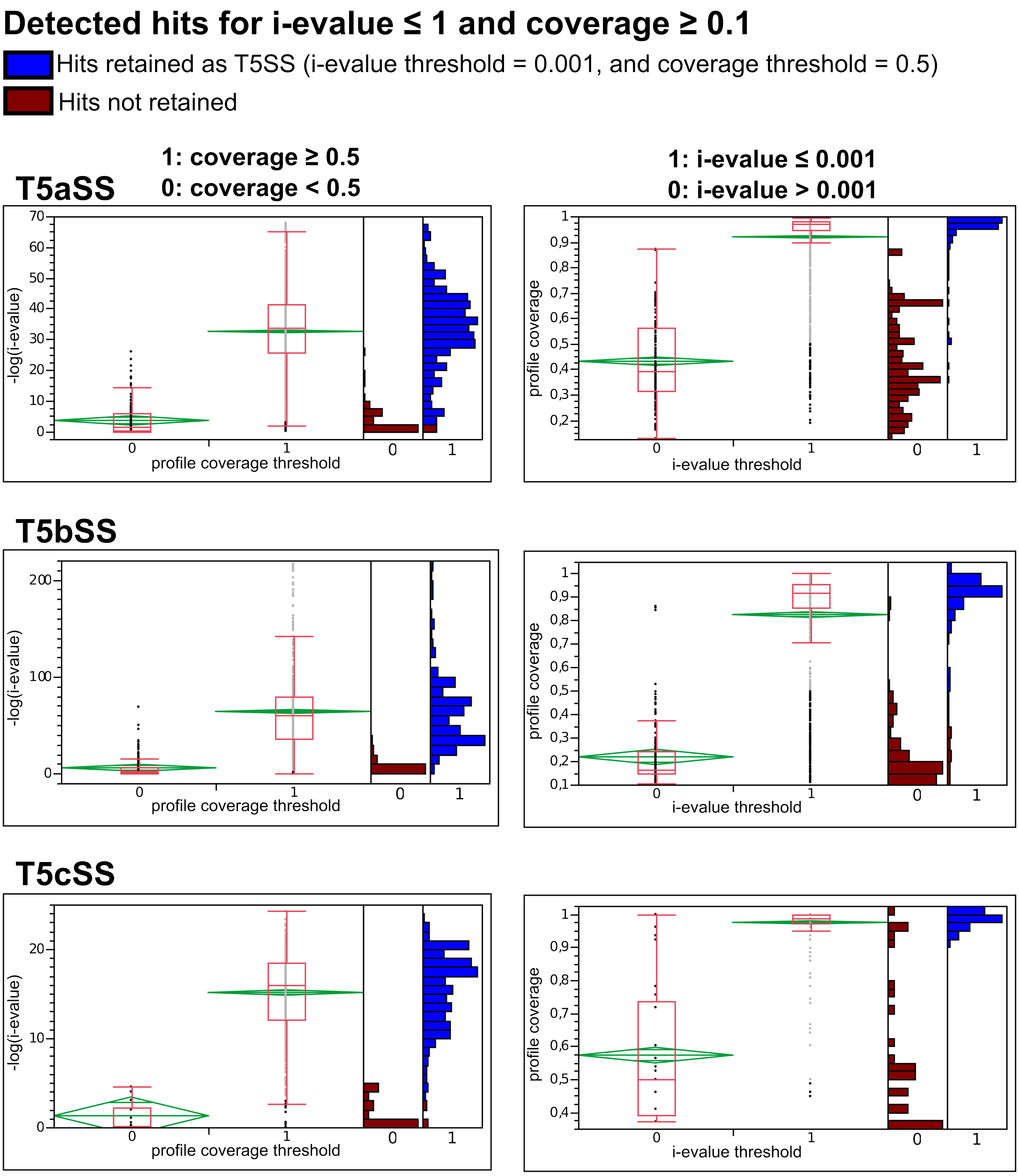

Figure S3. Analyses to define the parameters for T5SS detection.

Since the identification of T5SS depended on a single profile, we made a careful study of the Hmmer search parameters that allowed their correct identification. We display the distribution of profile coverage (proportion of the profile aligned with the hit, left column) and i-evalue (right) of hits obtained with relaxed threshold values (i-evalue of 1 and profile coverage of 0.1). For each of the two parameters, i-evalue and profile coverage, we show the boxplots and corresponding distributions. Hits validated by the threshold are in category 1 and the remaining in category 0. We also

show the histograms of the distribution of values of category 1 (blue) and of category 0 (red). The i-evalue and the profile coverage are highly correlated for T5aSS, T5bSS, and T5cSS hits. Following the analysis of these results, we set an i-evalue of 10^{-3} and a coverage threshold of 0.5 in the search for T5SS.

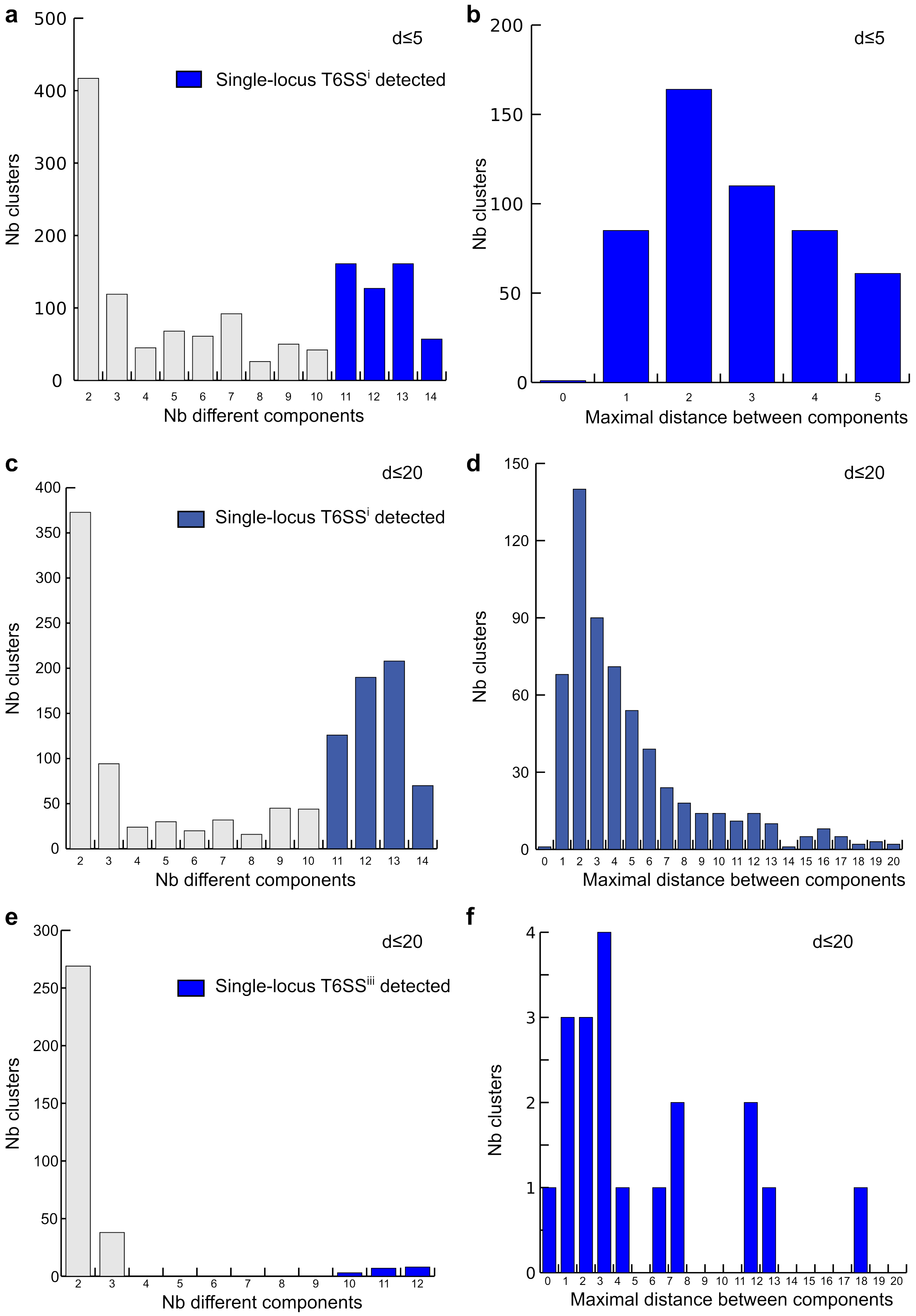

Figure S4. Analyses to define the parameters for T6SS^{i} and T6SS^{iii} detection.

a. Distribution of the number of different components of T6SS^{i} co-localized (d≤5). **b**. Distribution of the maximal distance observed between consecutive components in a

$T6SS^i$ detected with d≤5. This distribution suggests that larger values of *d* might improve the detection of $T6SS^i$. **c and d** panels show the same analyses with d≤20. The tail of the distribution of the maximal observed distance suggests that d≤20 misses fewer systems than d≤5 (also visible when comparing panels **c** and **a**). The minimal number of components required for a $T6SS^i$ was set to 11 in the $T6SS^i$ model, which corresponds to the second peak in the distribution in panel **c**. $T6SS^i$ detected as full systems with both values of the co-localization distance parameters are coloured in blue. **e** and **f** panels show the same analyses as in panels **a** and **b** for the system $T6SS^{iii}$. According to these graphs, the minimal number of components required for a $T6SS^{iii}$ was set to 8, and the co-localization parameter *d* was set to 20.

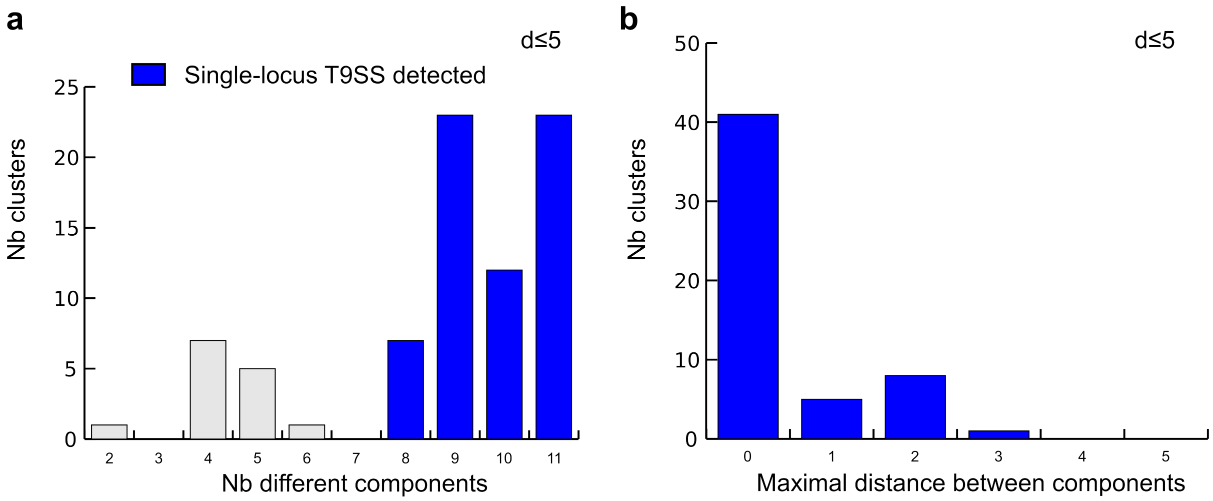

Figure S5. Analyses to define the parameters for T9SS detection.

a. Distribution of the number of different T9SS components co-localized (d≤5). **b**. Distribution of the maximal distance observed between consecutive components in a T9SS detected with d≤5. Based on these distributions, we set up a minimal number of components of 7 to define a complete T9SS, and a co-localization parameter *d* of 5 between T9SS components.

Table S1. Reference dataset: experimentally validated systems used to build HMM protein profiles for bacterial secretion systems and related appendages.

	Strain	Systems information	References
T1SS [2-4]	*Escherichia coli* K12 MG1665	Colicin V secretion (CvaB+CvaA+TolC)	[5]
	Escherichia coli K12 plasmid pHly152	Hemolysin secretion (HlyB+HlyD+TolC) HlyB and D were taken from the chromosome, TolC from the plasmid	[6]
	Erwinia chrysanthemi strain B374 clone pRUW1	Metalloprotease secretion (PrtD+PrtE+PrtF)	[7]
	Bordetella pertussis strain 18323	Cyclolysin secretion (CyaBDE)	[8]
	Serratia marcescens strain SM365	Hemophore HasA secretion (HasD+HasE+HasF)	[9-11]
	Serratia marcescens strain Sr41	Lipase secretion (LipB+LipC+LipD)	[12]
	Rhizobium leguminosarum strain 8401 subsp. viciae	Glucanase/Nodulation factor secretion (prsD+prsE)	[13]
	Caulobacter crescentus CB15	S-layer protein secretion (RsaD+RsaE+RsaF)	[14]
T2SS [15]	*Geobacter sulfurreducens* PCA		[16]
	Burkholderia pseudomallei K96243		[17]
	Caulobacter crescentus CB15		[18]
	Escherichia coli str. K-12 substr. MG1655		[19]
	Pseudomonas aeruginosa PAO1	Two systems: xcp and hxc	[20]
	Ralstonia solanacearum GMI1000		[21]
	Xanthomonas campestris pv. campestris str. ATCC 33913	Two systems: xps and xcs systems	[22,23]
T4P [24]	*Haemophilus influenzae* 86-028NP	Involved in DNA uptake	[25]
	Francisella tularensis subsp. tularensis SCHU S4	pilF missing	[26]
	Myxococcus xanthus DK 1622	pilE, pilF missing	[27]
	Neisseria meningitidis 8013		[28]
	Nostoc punctiforme PCC 73102		[29]
	Pseudomonas aeruginosa PAO1	pilC missing	[30]
	Ralstonia solanacearum GMI1000		[31]
	Thermus thermophilus HB27	pilE missing. Involved in DNA uptake	[32]
Tad [33]	*Aggregatibacter actinomycetemcomitans* CU1000N	Genbank: AY157714	[34,35]
	Caulobacter crescentus CB15	GenBank: AF229646.1	[36]
	Haemophilus ducreyi 35000H		[37]
	Pseudomonas aeruginosa PAO1		[38]
	Ralstonia solanacearum GMI1000 megaplasmid pGMI1000MP		[39]
	Yersinia enterocolitica subsp. enterocolitica 8081		[40]
T3SS			[41]
Flagellum			[41]
T4SS			[42]
T5aSS		PFAM03797, Beta domain (C-terminal translocator domain)	[43]
T5bSS [44]	*Haemophilus ducreyi* 35000HP	FHA secretion, LspB	[45]
	Edwardsiella tarda MZ8901	EthB (GI:2244626), cytolysin	[46,47]
	Pseudomonas aeruginosa PAO1	LepA (passenger) LepB (transporter) (TpsB4, PA4540-PA4541), protease	[48]
	Pseudomonas putida KT2440	HlpB (PP1450)	[49]
	Bordetella pertussis Tohama I	FhaC (Filamentous haemagglutinin - FHA export) adhesin (GI: 462082**)**	[50]
	Serratia marcescens	ShlB, cytolysin (haemolytic) (HlyB) (GI: 123205)	[51]
T5cSS		PFAM03895, C-terminal membrane-anchor domain	[43]
T6SS [52]	*Aeromonas hydrophila* subsp. hydrophila, strain ATCC 7966/NCIB 9240		[53]
	Burkholderia mallei strain ATCC 23344	System on chromosome 2	[54]

	Burkholderia pseudomallei strain K96243	System on chromosome 2	55
	Pseudomonas aeruginosa strain PAO1		56
	Pseudomonas syringae pv. syringae B728a		57
	Vibrio cholerae serovar O1, strain ATCC 39315/El Tor Inaba N16961	System on chromosome 2	58
	Dechloromonas aromatica RCB	No published experimental validation	52
	Escherichia coli CFT073		59
	Vibrio parahaemolyticus RIMD 2210633	T6SS-2, system on chromosome 2	60
T6SS[ii]	*Francisella tularensis subsp. tularensis* SCHU S4	T6SS FPI (locus 1, FTT1699-FTT1715)	61,62
T6SS[iii]	*Flavobacterium johnsoniae* UW101		63
	Bacteroidetes fragilis NCTC 9343		63
	Prevotella veroralis F0319		63
T9SS	*Flavobacterium johnsoniae* UW101		64
	Porphyromonas gingivalis ATCC 33277		65

Table S2. Validation dataset: detection of experimentally validated systems.

NB: When no strain name was available in the references, or when corresponding sequences were not available, we checked in all complete genomes available for the species ("Species-level" validation). Otherwise, we performed the detection on the same strain ("Strain-level" validation).

	Strain	Detection	Comments	References
**T1SS**	*Pseudomonas aeruginosa* PAO1	"Strain-level" validation.<br><br>2/2 detected (+1 but no experimental evidence)	3 complete systems detected (abc+mfp+omf):<br>- aprDEF system (PA1246-1248, next to the secreted protein aprA, PA1249)<br>- PA1875-1877 (no substrate reported)<br>- hasA (PA3407) secreting system (PA3404-3406 hasED system)	[66,67]
**T1SS**	*E. coli* CFT073	"Strain-level" validation.<br><br>1/1 detected	TosA RTX secreted protein.	[59]
**T1SS**	*Bordetella pertussis* Tohama I	"Strain-level" validation.<br><br>1/1 detected	CyaBDE involved in the secretion of the cyclolysin RTX protein. In one locus.	[68,69]
**T1SS**	*Salmonella enterica* Typhimurium ATCC14028S	"Strain-level" validation.<br><br>1/1 detected	Secretion of the giant non-fimbrial adhesin siiE. In one locus.	[70]
**T1SS**	*Serratia marcescens* FGI94	"Species-level" validation<br><br>1/1 detected	Secretion of the hemophore HasA by HasDE/TolC system (scattered, omf in trans)	[10,71]
**T2SS**	*Escherichia coli* pO157 plasmid	"Strain-level" validation<br><br>1/1 detected	Secretion of the StcE zinc mettaloprotease, a host-cell adherence factor	[72,73]
**T2SS**	*Escherichia coli* ETEC H10407	"Strain-level" validation<br><br>1/1 detected		[74,75]
**T2SS**	*Klebsiella pneumoniae* strain K21	"Species-level" validation.<br><br>(6/6 complete genomes available)<br>1/1 detected	T2SS are detected in 6 strains genomes (gspC missing in 5/6, gspH in 6/6)	[76]
**T2SS**	*Klebsiella oxytoca*	"Species-level" validation.<br><br>(2/2 complete genomes available)<br>2/2 detected	- strain KCTC 1686: 2 complete systems (gspH and gspC missing in both)<br>- strain E718: 2 complete systems gspHCNL missing in 1/2, gspH only in 1/2	[77]
**T2SS**	*Dickeya dadantii* (*Erwinia chrysanthemi)*	Species"-level validation?<br><br>2 T2SS. From references: 1 complete "Out" system with 14 proteins, 1 with gspBHO missing "Stt"<br><br>1/2 detected	- 1 complete detected (H,N missing)<br>- 1 partial (stt) not detected because C, K, M hits are matched but do not pass the hits filtering (i-evalue and coverage)	[77-79]
**T2SS**	*Erwinia carotovora*	Species"-level validation.<br><br>(2/2 complete genomes available)<br>1/1 detected	1 full system found in each sequenced genomes (strains subsp. carotovorum PC1 and PCC21)	[77]
**T2SS**	*Legionella pneumophila*	T2SS scattered in 5 loci. Unusual system:	- 1 system partially detected in strain subsp. pneumophila str. Philadelphia 1 (system in 2 loci)	[80,81]

		C alone, LM together (not detected), DE together and FGHIJK together. Moreover, exists recombination within one of the locus.<br><br>“Species-level” validation, but unusual system architecture => not detected<br>0/1 detected	- Another partially detected for 6 other strains (MCNOL missing: MCO detected and extracted, but L below coverage, N not detected)	
**T2SS**	*Yersinia enterocolitica* subsp. enterocolitica 8081	2 kinds of T2SS, one widespread, Yts2 (cf. *Y. pestis*), the other linked to high-pathogenicity group: Yts1.<br>Yts1 "full", Yts2 with (S)MLH missing.<br><br>“Strain-level” validation for 1 system<br>1/2 detected	- Yts1 system detected (HCN missing)<br>- Yts2 partially detected (MLHKCN missing, but normal for MLH. K and C matched but not retained after filtering (i-evalue), N is not found	[82]
**T2SS**	*Pseudoalteromonas haloplanktis* TAC125	“Strain-level” validation<br>1/1 detected	Full system detected.	[83]
**T2SS**	*Pseudoalteromonas ruthenica* CP76	“Strain-level” validation<br>1/1 detected	Full system detected. Except GspG: the corresponding ORF was not predicted.	[84,85]
**T2SS**	*Shewanella oneidensis* MR-1	“Strain-level” validation<br>1/1 detected	Full system detected.	[86]
**T2SS**	*Vibrio cholerae* O1 biovar El Tor str. N16961	“Strain-level” validation<br>1/1 detected	Full system detected. Except GspD: the corresponding ORF was not predicted.	[87,88]
**T2SS**	*Vibrio vulnificus* CMCP6	“Strain-level” validation<br>1/1 detected	Full system detected. Except GspG.	[89]
**T2SS**	*Stenotrophomonas maltophilia* K279a	0/1 detected	Xps-type system, not completely detected. GspH, GspJ, GspM, GspN not retained in cluster. PilN/PilAE profiles matched resp. GspL/GspH proteins. No match for GspM and GspN. GspJ was a poor hit not retained after filtering.	[90]
**T2SS**	*Pseudomonas aeruginosa* strain PA7	“Strain-level” validation<br>1/1 detected	Full system detected. Except GspC, GspO.	[83]
**T4P**	*Vibrio cholerae* O1 biovar El Tor str. N16961	8/8 genomes have this T4P<br><br>“Strain-level” validation<br>1/1 detected	Complete T4P found on multiple loci.	[91]
**T4P**	*Acidovorax avenae* subsp. citrulli M6	“Species-level” validation 1/1 detected	Complete T4P found on multiple loci.	[92]
**T4P**	*Verminephrobacter eiseniae* strain EF01-2 (genome)	Experimental control in strain EF05-2r<br><br>“Species-level” validation<br>1/1 detected	Complete T4P found on multiple loci.	[93]
**T4P**	*Aeromonas salmonicida* A449	“Strain-level” validation<br>1/1 detected	Complete T4P found on multiple loci.	[94]
**Tad**	*Eubacterium rectale*	“Species-level” validation<br>1/1 detected	TadZ not retained after hits filtering	[95]
**Tad**	*Pectobacterium wasabiae* WPP163	“Species-level” validation<br>1/1 detected		[96]

**Tad**	*Aeromonas salmonicida* A449	"Strain-level" validation<br>1/1 detected		94
**T3SS**				41
**Flagellum**				41
**T4SS**				42
**T5aSS**	*Pseudomonas aeruginosa* PAO1	PA5112 EstA, esterase<br><br>"Strain-level" validation<br>1/1 detected		66
**T5aSS**	*Pseudomonas aeruginosa*	PA0328 autotransporter, aaaA<br><br>"Strain-level" validation<br>1/1 detected		97
**T5aSS**	*Escherichia coli* CFT073	*E. coli* Ag43 a and b, 2 adhesins<br><br>"Strain-level" validation<br>1/1 detected		98,99
**T5aSS**	*Bordetella pertussis* Tohama I	Pertactin (prn), adhesin<br><br>"Strain-level" validation<br>1/1 detected	BP1054 sequence identifier in the genome	100
**T5bSS**	*Escherichia coli* 536	CdiB protein which facilitates CdiA secretion, for CDI.<br><br>"Strain-level" validation<br>1/1 detected		101
**T5bSS**	*Escherichia coli* O157:H7 EDL933	OtpB<br><br>"Strain-level" validation<br>1/1 detected		102
**T5bSS**	*Pseudomonas aeruginosa* PAO1	PA4082 CupB5 "hemmaglutinin-like"<br><br>0/1 detected	Not found. Part of a chaperone usher pathway locus. Atypical T5bSS, see Text S1.	66,103
**T5cSS**	*Escherichia coli* EDL933	EhaG positional ortholog of UpaG<br><br>"Strain-level" validation<br>1/1 detected		104
**T5cSS**	*Escherichia coli* CFT073	UpaG, adhesin<br><br>"Strain-level" validation<br>1/1 detected		105
**T6SS**[i]	*Edwardsiella tarda* PPD130/91	"Species-level" validation 1/1 detected	Found in 2/2 genomes (TssAMJ missing)	106
**T6SS**[i]	*Acinetobacter baumannii* strain AYE	"Strain-level" validation<br>1/1 detected		107
**T6SS**[i]	Citrobacter rodentium CTS1	"Species-level" validation<br>1/1 detected	Exists multiple systems	108
**T6SS**[i]	*Agrobacterium tumefaciens* C58	T6SS acid-induced<br><br>"Strain-level" validation<br>1/1 detected	TssAJ missing	109
**T6SS**[i]	*Burkholderia cenocepacia* K56-2	"Species or strain?"-level validation<br>1/1 detected	Profiles matched from BCAL0337 to BCAL0351 (BCAL0333 -BCAL0352 in the paper)	110

**T6SS**[i]	*Yersinia pestis*	"Species-level" validation 1/1 detected	System detected but TssA missing (TssJ missing in the paper as well)	111
**T6SS**[i]	*Escherichia coli* O42	"Strain-level" validation<br><br>1/2 detected	A complete T6SS is detected (sci-1) followed by a second uncomplete (TssMJDEA missing, sci-2).	112

Table S3. Distribution of protein secretion systems in the analysed dataset.

See the file TableS3.xls attached to the manuscript.

Table S4. List of the HMM protein profiles included in the study.

Hmmer profile name	System	Profile length	Nb sequences	Origin
**T1SS_abc.hmm**	T1SS	501	8	This study
**T1SS_mfp.hmm**	T1SS	378	8	This study
**T1SS_omf.hmm**	T1SS	400	7	This study
**T2SS_gspC.hmm**	T2SS	76	9	This study
**T2SS_gspD.hmm**	T2SS	596	10	This study
**T2SS_gspE.hmm**	T2SS	378	10	This study
**T2SS_gspF.hmm**	T2SS	394	10	This study
**T2SS_gspG.hmm**	T2SS	131	10	This study
**T2SS_gspH.hmm**	T2SS	153	3	This study
**T2SS_gspI.hmm**	T2SS	109	9	This study
**T2SS_gspJ.hmm**	T2SS	123	8	This study
**T2SS_gspK.hmm**	T2SS	247	10	This study
**T2SS_gspL.hmm**	T2SS	254	9	This study
**T2SS_gspM.hmm**	T2SS	134	7	This study
**T2SS_gspN.hmm**	T2SS	217	137	This study
**T2SS_gspO.hmm**	T2SS	233	3	This study
**T4P_pilAE.hmm**	T4P	165	25	This study
**T4P_pilB.hmm**	T4P	383	8	This study
**T4P_pilC.hmm**	T4P	339	7	This study
**T4P_pilD.hmm**	T4P	232	8	This study
**T4P_pilM.hmm**	T4P	338	6	This study
**T4P_pilN.hmm**	T4P	193	4	This study
**T4P_pilO.hmm**	T4P	187	5	This study
**T4P_pilP.hmm**	T4P	143	4	This study
**T4P_pilQ.hmm**	T4P	279	8	This study
**Tad_flp.hmm**	Tad	56	8	This study
**Tad_rcpA.hmm**	Tad	405	6	This study
**Tad_tadA.hmm**	Tad	369	6	This study
**Tad_tadB.hmm**	Tad	220	6	This study
**Tad_tadC.hmm**	Tad	295	6	This study
**Tad_tadD.hmm**	Tad	237	3	This study
**Tad_tadE.hmm**	Tad	152	3	This study
**Tad_tadF.hmm**	Tad	196	3	This study
**Tad_tadV.hmm**	Tad	152	6	This study
**Tad_tadZ.hmm**	Tad	416	6	This study
**T3SS_sctC.hmm**	T3SS	544	6	[41]
**T3SS_sctJ.hmm**	T3SS	250	7	[41]
**T3SS_sctN.hmm**	T3SS	434	7	[41]
**T3SS_sctQ.hmm**	T3SS	69	7	[41]
**T3SS_sctR.hmm**	T3SS	199	7	[41]
**T3SS_sctS.hmm**	T3SS	81	7	[41]
**T3SS_sctT.hmm**	T3SS	255	7	[41]
**T3SS_sctU.hmm**	T3SS	337	7	[41]
**T3SS_sctV.hmm**	T3SS	668	7	[41]
**Flg_flgB.hmm**	Flg	129	8	[41]
**Flg_flgC.hmm**	Flg	141	8	[41]

**Flg_fliE.hmm**	Flg	102	8	[41]
**Flg_sctJ_FLG.hmm**	Flg	516	8	[41]
**Flg_sctN_FLG.hmm**	Flg	430	8	[41]
**Flg_sctQ_FLG.hmm**	Flg	75	10	[41]
**Flg_sctR_FLG.hmm**	Flg	202	8	[41]
**Flg_sctS_FLG.hmm**	Flg	89	8	[41]
**Flg_sctT_FLG.hmm**	Flg	259	6	[41]
**Flg_sctU_FLG.hmm**	Flg	344	7	[41]
**Flg_sctV_FLG.hmm**	Flg	671	8	[41]
**T4SS_t4cp1.hmm**	T4SS	110	253	[42,113]
**T4SS_t4cp2.hmm**	T4SS	222	253	[42,113]
**T4SS_tcpA.hmm**	T4SS	464	25	[42,113]
**T4SS_virb4.hmm**	T4SS	943	182	[42,113]
**T4SS_MOBB.hmm**	T4SS	224	58	[42,113]
**T4SS_MOBC.hmm**	T4SS	211	17	[42,113]
**T4SS_MOBF.hmm**	T4SS	880	65	[42,113]
**T4SS_MOBH.hmm**	T4SS	204	17	[42,113]
**T4SS_MOBP1.hmm**	T4SS	233	144	[42,113]
**T4SS_MOBP2.hmm**	T4SS	509	6	[42,113]
**T4SS_MOBP3.hmm**	T4SS	308	2	[42,113]
**T4SS_MOBQ.hmm**	T4SS	217	66	[42,113]
**T4SS_MOBT.hmm**	T4SS	366	160	[42,113]
**T4SS_MOBV.hmm**	T4SS	204	56	[42,113]
**T4SS_B_traE.hmm**	T4SS_B	116	17	[42,113]
**T4SS_B_traF.hmm**	T4SS_B	101	16	[42,113]
**T4SS_B_traH.hmm**	T4SS_B	117	8	[42,113]
**T4SS_B_traI.hmm**	T4SS_B	209	13	[42,113]
**T4SS_B_traJ.hmm**	T4SS_B	341	16	[42,113]
**T4SS_B_traK.hmm**	T4SS_B	207	18	[42,113]
**T4SS_B_traL.hmm**	T4SS_B	96	10	[42,113]
**T4SS_B_traM.hmm**	T4SS_B	450	13	[42,113]
**T4SS_B_traN.hmm**	T4SS_B	304	16	[42,113]
**T4SS_B_traO.hmm**	T4SS_B	192	12	[42,113]
**T4SS_B_traP.hmm**	T4SS_B	296	5	[42,113]
**T4SS_B_traQ.hmm**	T4SS_B	139	12	[42,113]
**T4SS_C_alr7204.hmm**	T4SS_C	195	12	[42,113]
**T4SS_C_alr7205.hmm**	T4SS_C	110	12	[42,113]
**T4SS_C_alr7207.hmm**	T4SS_C	239	5	[42,113]
**T4SS_C_alr7208.hmm**	T4SS_C	353	12	[42,113]
**T4SS_C_alr7209.hmm**	T4SS_C	252	18	[42,113]
**T4SS_C_alr7210.hmm**	T4SS_C	525	5	[42,113]
**T4SS_C_alr7211.hmm**	T4SS_C	248	8	[42,113]
**T4SS_C_alr7212.hmm**	T4SS_C	399	11	[42,113]
**T4SS_F_traB.hmm**	T4SS_F	431	8	[42,113]
**T4SS_F_traE.hmm**	T4SS_F	193	73	[42,113]
**T4SS_F_traF.hmm**	T4SS_F	276	9	[42,113]
**T4SS_F_traG.hmm**	T4SS_F	948	50	[42,113]
**T4SS_F_traH.hmm**	T4SS_F	461	65	[42,113]
**T4SS_F_traK.hmm**	T4SS_F	278	74	[42,113]

**T4SS_F_traL.hmm**	T4SS_F	100	62	[42,113]
**T4SS_F_traN.hmm**	T4SS_F	655	73	[42,113]
**T4SS_F_traU.hmm**	T4SS_F	334	76	[42,113]
**T4SS_F_traV.hmm**	T4SS_F	245	69	[42,113]
**T4SS_F_traW.hmm**	T4SS_F	212	75	[42,113]
**T4SS_F_trbC.hmm**	T4SS_F	229	62	[42,113]
**T4SS_G_tfc10.hmm**	T4SS_G	120	61	[42,113]
**T4SS_G_tfc11.hmm**	T4SS_G	130	61	[42,113]
**T4SS_G_tfc12.hmm**	T4SS_G	221	49	[42,113]
**T4SS_G_tfc13.hmm**	T4SS_G	287	74	[42,113]
**T4SS_G_tfc14.hmm**	T4SS_G	478	74	[42,113]
**T4SS_G_tfc15.hmm**	T4SS_G	138	49	[42,113]
**T4SS_G_tfc17.hmm**	T4SS_G	133	14	[42,113]
**T4SS_G_tfc18.hmm**	T4SS_G	114	49	[42,113]
**T4SS_G_tfc19.hmm**	T4SS_G	509	65	[42,113]
**T4SS_G_tfc2.hmm**	T4SS_G	202	49	[42,113]
**T4SS_G_tfc22.hmm**	T4SS_G	457	49	[42,113]
**T4SS_G_tfc23.hmm**	T4SS_G	331	49	[42,113]
**T4SS_G_tfc24.hmm**	T4SS_G	140	56	[42,113]
**T4SS_G_tfc3.hmm**	T4SS_G	240	53	[42,113]
**T4SS_G_tfc5.hmm**	T4SS_G	172	63	[42,113]
**T4SS_G_tfc7.hmm**	T4SS_G	249	66	[42,113]
**T4SS_G_tfc8.hmm**	T4SS_G	116	65	[42,113]
**T4SS_G_tfc9.hmm**	T4SS_G	79	57	[42,113]
**T4SS_I_traE.hmm**	T4SS_I	274	9	[42,113]
**T4SS_I_traI.hmm**	T4SS_I	273	23	[42,113]
**T4SS_I_traK.hmm**	T4SS_I	87	17	[42,113]
**T4SS_I_traL.hmm**	T4SS_I	156	17	[42,113]
**T4SS_I_traM.hmm**	T4SS_I	229	25	[42,113]
**T4SS_I_traN.hmm**	T4SS_I	336	25	[42,113]
**T4SS_I_traO.hmm**	T4SS_I	441	21	[42,113]
**T4SS_I_traP.hmm**	T4SS_I	239	17	[42,113]
**T4SS_I_traQ.hmm**	T4SS_I	179	17	[42,113]
**T4SS_I_traR.hmm**	T4SS_I	134	14	[42,113]
**T4SS_I_traT.hmm**	T4SS_I	266	9	[42,113]
**T4SS_I_traU.hmm**	T4SS_I	1008	24	[42,113]
**T4SS_I_traV.hmm**	T4SS_I	204	10	[42,113]
**T4SS_I_traW.hmm**	T4SS_I	405	21	[42,113]
**T4SS_I_traY.hmm**	T4SS_I	745	23	[42,113]
**T4SS_I_trbA.hmm**	T4SS_I	413	19	[42,113]
**T4SS_I_trbB.hmm**	T4SS_I	347	16	[42,113]
**T4SS_T_virB1.hmm**	T4SS_T	143	36	[42,113]
**T4SS_T_virB10.hmm**	T4SS_T	375	71	[42,113]
**T4SS_T_virB11.hmm**	T4SS_T	328	73	[42,113]
**T4SS_T_virB2.hmm**	T4SS_T	113	171	[42,113]
**T4SS_T_virB3.hmm**	T4SS_T	81	132	[42,113]
**T4SS_T_virB5.hmm**	T4SS_T	196	110	[42,113]
**T4SS_T_virB6.hmm**	T4SS_T	433	149	[42,113]
**T4SS_T_virB8.hmm**	T4SS_T	229	151	[42,113]

**T4SS_T_virB9.hmm**	T4SS_T	269	151	[42,113]
**T5aSS_PF03797.hmm**	T5aSS	265	212	PFAM profile
**T5bSS_translocator.hmm**	T5bSS	533	6	This study
**T5cSS_PF03895.hmm**	T5cSS	78	110	PFAM profile
**T6SS_evpJ.hmm**	T6SS	95	7	This study
**T6SS_tssA.hmm**	T6SS	435	10	This study
**T6SS_tssB.hmm**	T6SS	163	7	This study
**T6SS_tssC.hmm**	T6SS	486	8	This study
**T6SS_tssD.hmm**	T6SS	170	6	This study
**T6SS_tssE.hmm**	T6SS	146	7	This study
**T6SS_tssF.hmm**	T6SS	602	6	This study
**T6SS_tssG.hmm**	T6SS	332	6	This study
**T6SS_tssH.hmm**	T6SS	872	7	This study
**T6SS_tssI.hmm**	T6SS	603	11	This study
**T6SS_tssJ.hmm**	T6SS	154	7	This study
**T6SS_tssK.hmm**	T6SS	442	7	This study
**T6SS_tssL.hmm**	T6SS	214	8	This study
**T6SS_tssM.hmm**	T6SS	1111	7	This study
**T6SSii_dotU.hmm**	T6SSii	205	30	This study
**T6SSii_iglA.hmm**	T6SSii	183	32	This study
**T6SSii_iglB.hmm**	T6SSii	471	500	This study
**T6SSii_iglC.hmm**	T6SSii	207	30	This study
**T6SSii_iglD.hmm**	T6SSii	396	30	This study
**T6SSii_iglE.hmm**	T6SSii	123	30	This study
**T6SSii_iglF.hmm**	T6SSii	553	27	This study
**T6SSii_iglG.hmm**	T6SSii	171	29	This study
**T6SSii_iglH.hmm**	T6SSii	474	29	This study
**T6SSii_iglI.hmm**	T6SSii	381	29	This study
**T6SSii_iglJ.hmm**	T6SSii	236	30	This study
**T6SSii_pdpA.hmm**	T6SSii	818	31	This study
**T6SSii_pdpB.hmm**	T6SSii	1091	30	This study
**T6SSii_pdpC.hmm**	T6SSii	1326	27	This study
**T6SSii_pdpD.hmm**	T6SSii	1193	24	This study
**T6SSii_pdpE.hmm**	T6SSii	190	26	This study
**T6SSii_vgrG.hmm**	T6SSii	162	29	This study
**T6SSiii_tssB.hmm**	T6SSiii	147	3	This study
**T6SSiii_tssC.hmm**	T6SSiii	459	3	This study
**T6SSiii_tssD.hmm**	T6SSiii	121	7	This study
**T6SSiii_tssE.hmm**	T6SSiii	143	3	This study
**T6SSiii_tssF.hmm**	T6SSiii	599	3	This study
**T6SSiii_tssG.hmm**	T6SSiii	315	3	This study
**T6SSiii_tssH.hmm**	T6SSiii	818	3	This study
**T6SSiii_tssI.hmm**	T6SSiii	596	3	This study
**T6SSiii_tssK.hmm**	T6SSiii	384	3	This study
**T6SSiii_tssN.hmm**	T6SSiii	272	3	This study
**T6SSiii_tssP.hmm**	T6SSiii	241	3	This study
**T6SSiii_tssQ.hmm**	T6SSiii	790	2	This study
**T9SS_porQ.hmm**	T9SS	332	40	This study
**T9SS_porU.hmm**	T9SS	1110	52	This study

**T9SS_porV.hmm**	T9SS	382	62	This study
**T9SS_sprE.hmm**	T9SS	713	59	This study
**T9SS_sprT.hmm**	T9SS	236	39	This study
**T9SS_gldJ_TIGR03524.hmm**	T9SS	559	3	TIGRFAM profile
**T9SS_gldK_TIGR03525.hmm**	T9SS	449	5	TIGRFAM profile
**T9SS_gldL_TIGR03513.hmm**	T9SS	247	5	TIGRFAM profile
**T9SS_gldM_TIGR03517.hmm**	T9SS	524	7	TIGRFAM profile
**T9SS_gldN_TIGR03523.hmm**	T9SS	280	7	TIGRFAM profile
**T9SS_sprA_PF14349.hmm**	T9SS	426	88	PFAM profile
**T9SS_sprA-2_PF12118.hmm**	T9SS	295	40	PFAM profile
**T9SS_sprA-3_TIGR04189.hmm**	T9SS	2319	15	TIGRFAM profile

Table S5. Methods employed to build HMM protein profiles.

System	Clustering	Alignment	Nb profiles
**T1SS**	None. Use of protein families from the ABCISSE database [3,4]	Muscle (version 3.8.31) default parameters, then manual edition in Seaview [114,115]	3
**T2SS**	BLASTp version 2.2.19 "all against all", followed by MCL clustering [116] on -log(Evalue) with an Evalue threshold set to e^{-15} (MCL inflation parameter I=1.45 for all protein families except GspC/GspK: I=1 and GspG: I=1.7).<br>GspN was built on a wider dataset (based on the Silix clustering of contiguous genes matching the other T2SS profiles with the following parameters: identity ≥ 20%, sequence overlap ≥ 50%, and sequence length ≥ 30, *i.e.* 137 sequences [117])	T-COFFEE Version_9.02.r1228 (Expresso when 3D structure available) [118]<br>PDB identifiers for 3D templates:<br>GspE: 1p9wA<br>GspI: 3ci0I<br>GspJ: 3ci0J<br>GspJ: 3njeB<br>GspK: 3ci0K<br>GspG: 3fu1B<br>GspG: 3g20B<br>For GspN, the Mafft program was used (MAFFT v6.843b, with "auto" strategy for alignment [119]).	13
**T4P**	BLASTp version 2.2.19 "all against all", then MCL clustering on -log(Evalue) with an Evalue threshold set to e^{-15} (MCL inflation parameter I=1.2 except for pilB and pilT_pilU: I=2 [116])	Muscle (version 3.8.31) default parameters, then manual edition in Seaview [114,115]	11
**Tad**	BLASTp version 2.2.19 "all against all", then MCL clustering on -log(Evalue) with an Evalue threshold set to e^{-15} (MCL inflation parameter I=1.5 for all except RcpA, TadC, TadV and TadZ: I=1 [116])	Muscle (version 3.8.31) default parameters, then manual edition in Seaview [114,115]	10
**T3SS**	[41]	[41]	9
**Flagellum**	[41]	[41]	11
**T4SS**	[42,113]	[42,113]	90
**T5aSS**	Profile from PFAM [43,120]	profile from PFAM [43,120]	1
**T5bSS**		T-COFFEE Version_9.03.r1542 (One 3D structure found, PDB 2qdzA: Expresso algorithm) then manual edition with Seaview [115,118]	1
**T5cSS**	Profile from PFAM [43,120]	profile from PFAM [43,120]	1
**T6SS**	BLASTp version 2.2.19 "all against all", then MCL clustering on -log(Evalue) with an Evalue threshold set to e^{-15} (MCL inflation parameter I=1.6)	Muscle (version 3.8.31) default parameters, then manual edition in Seaview [114,115]	14
**T6SS[ii]**	BLASTp version 2.2.30 using *Francisella tularensis* FPI system as a query against the whole genome dataset: hits with Evalue below e^{-20} were retained. Then Blastp "all against all", and MCL clustering on -log(Evalue) with an Evalue threshold set to e^{-6} (MCL inflation parameter I=2) to build the protein families	Mafft (version v7.215) 'auto' mode, default parameters, then automatic edition with a home-made script using BMGE (version 1.1), (Blosum 30 matrix, 20% of gaps allowed) [121,122] to trim the extremities of the alignment	
**T6SS[iii]**	BLASTp version 2.2.30 "all against all", then MCL clustering on -log(Evalue) with an Evalue threshold set to e^{-6} (MCL inflation parameter I=2.5)	Mafft (version v7.215) 'auto' mode, default parameters, then manual edition in Seaview [115,121]	12
**T9SS**	For PorU, PorV, SprE and SprT, BLASTp (version 2.2.30) was run from *F. johnsoniae* sequences against the whole genome dataset, and hits with Evalue below e^{-50}, e^{-50}, e^{-40} and e^{-15} resp. were retained as family members. As no *F. johnsoniae* homolog was present in the T9SS, the *P. gingivalis* sequence was used in the same way for PorQ, with an Evalue threshold of e^{-20}.<br>Other profiles were obtained from PFAM or TIGRFAM (see Table S4)	Mafft (version v7.215) 'auto' mode, default parameters, then automatic edition with a home-made script using BMGE (version 1.1), (Blosum 30 matrix, 20% of gaps allowed) [121,122] to trim the extremities of the alignment	13

Table S6. TIGRFAM profiles matching the TXSScan profiles.

The analysis was done by searching for homology between each protein profile and TIGRFAM (downloaded the 10 January 2016). We used hhmake v2.0.15 [123] using default parameters to produce the protein profiles using the seed alignments for TXSScan and TIGRFAM. The profiles were then aligned with hhsearch v2.0.15 [123] with default parameters from the same package. Hits with Prob>0.98 were included in the table.

Profile	#hit	TIGFAM profile	Prob	E-value
**FLG_flgB**	1	TIGR01396	100	1.20E-39
**FLG_flgC**	1	TIGR01395	100	3.60E-57
**FLG_flgC**	2	TIGR02488	99.2	5.90E-16
**FLG_flgC**	3	TIGR03506	98.4	1.80E-11
**FLG_flgC**	4	TIGR02490	98.4	2.10E-11
**FLG_flhA_sctV**	1	TIGR01398	100	6.00E-255
**FLG_flhA_sctV**	2	TIGR01399	100	8.00E-168
**FLG_flhB_sctU**	1	TIGR00328	100	2.00E-132
**FLG_flhB_sctU**	2	TIGR01404	100	2.80E-89
**FLG_flhB_sctU**	3	TIGR00789	99.7	1.40E-22
**FLG_fliE**	1	TIGR00205	100	6.20E-38
**FLG_fliF_sctJ**	1	TIGR00206	100	5.00E-143
**FLG_fliF_sctJ**	2	TIGR02544	99.2	5.00E-16
**FLG_fliI_sctN**	1	TIGR01026	100	9.00E-199
**FLG_fliI_sctN**	2	TIGR03497	100	4.00E-193
**FLG_fliI_sctN**	3	TIGR03496	100	1.00E-189
**FLG_fliI_sctN**	4	TIGR03498	100	2.00E-169
**FLG_fliI_sctN**	5	TIGR02546	100	8.00E-168
**FLG_fliI_sctN**	6	TIGR01039	100	8.10E-52
**FLG_fliI_sctN**	7	TIGR03305	100	1.40E-46
**FLG_fliI_sctN**	8	TIGR00962	100	3.70E-45
**FLG_fliI_sctN**	9	TIGR01041	100	9.60E-45
**FLG_fliI_sctN**	10	TIGR03324	100	8.80E-44
**FLG_fliI_sctN**	11	TIGR01043	100	1.10E-41
**FLG_fliI_sctN**	12	TIGR01042	99.9	4.70E-34
**FLG_fliI_sctN**	13	TIGR01040	99.9	3.90E-33
**FLG_fliI_sctN**	14	TIGR00767	99.7	6.30E-22
**FLG_fliN-fliY_sctQ**	1	TIGR02480	100	9.10E-36
**FLG_fliN-fliY_sctQ**	2	TIGR02551	99.4	4.80E-18
**FLG_fliP_sctR**	1	TIGR01103	100	1.00E-104
**FLG_fliP_sctR**	2	TIGR01102	100	6.70E-57
**FLG_fliQ_sctS**	1	TIGR01402	100	5.30E-44
**FLG_fliQ_sctS**	2	TIGR01403	99.7	1.90E-23

**FLG_fliR_sctT**	1	TIGR01400	100	2.50E-59
**FLG_fliR_sctT**	2	TIGR01401	99.9	8.90E-32
**T1SS_abc**	1	TIGR01842	100	1.00E-185
**T1SS_abc**	2	TIGR01846	100	1.00E-164
**T1SS_abc**	3	TIGR03375	100	2.00E-111
**T1SS_abc**	4	TIGR03796	100	2.70E-87
**T1SS_abc**	5	TIGR02204	100	1.60E-86
**T1SS_abc**	6	TIGR03797	100	4.90E-83
**T1SS_abc**	7	TIGR00958	100	3.40E-80
**T1SS_abc**	8	TIGR02203	100	2.10E-77
**T1SS_abc**	9	TIGR01193	100	2.10E-76
**T1SS_abc**	10	TIGR02857	100	6.90E-67
**T1SS_abc**	11	TIGR02868	100	8.80E-58
**T1SS_abc**	12	TIGR01192	100	2.60E-54
**T1SS_abc**	13	TIGR04520	100	4.20E-42
**T1SS_abc**	14	TIGR00957	100	1.70E-41
**T1SS_abc**	15	TIGR02673	100	7.70E-41
**T1SS_abc**	16	TIGR04521	100	2.00E-40
**T1SS_abc**	17	TIGR02315	100	6.00E-37
**T1SS_abc**	18	TIGR00972	100	1.40E-36
**T1SS_abc**	19	TIGR02982	100	1.10E-35
**T1SS_abc**	20	TIGR01186	100	2.20E-35
**T1SS_abc**	21	TIGR00968	99.9	1.10E-33
**T1SS_abc**	22	TIGR03608	99.9	1.90E-33
**T1SS_abc**	23	TIGR01277	99.9	1.10E-32
**T1SS_abc**	24	TIGR03410	99.9	1.70E-32
**T1SS_abc**	25	TIGR01166	99.9	2.20E-32
**T1SS_abc**	26	TIGR03265	99.9	2.30E-32
**T1SS_abc**	27	TIGR03864	99.9	2.70E-32
**T1SS_abc**	28	TIGR02211	99.9	2.70E-32
**T1SS_abc**	29	TIGR02769	99.9	1.60E-31
**T1SS_abc**	30	TIGR02314	99.9	2.40E-31
**T1SS_abc**	31	TIGR04406	99.9	8.10E-31
**T1SS_abc**	32	TIGR01187	99.9	1.40E-30
**T1SS_abc**	33	TIGR03522	99.9	5.20E-30
**T1SS_abc**	34	TIGR01188	99.9	3.70E-29
**T1SS_abc**	35	TIGR00957	99.9	1.40E-28
**T1SS_abc**	36	TIGR03258	99.9	1.60E-28
**T1SS_abc**	37	TIGR03005	99.9	2.70E-28
**T1SS_abc**	38	TIGR01184	99.9	1.50E-27
**T1SS_abc**	39	TIGR02142	99.8	7.00E-27
**T1SS_abc**	40	TIGR01189	99.8	9.10E-27
**T1SS_abc**	41	TIGR00955	99.8	3.10E-26
**T1SS_abc**	42	TIGR01271	99.8	4.20E-26
**T1SS_abc**	43	TIGR03771	99.8	2.30E-25
**T1SS_abc**	44	TIGR03719	99.8	3.10E-25

**T1SS_abc**	45	TIGR03740	99.8	4.30E-25
**T1SS_abc**	46	TIGR01288	99.8	6.50E-25
**T1SS_abc**	47	TIGR02324	99.8	7.60E-25
**T1SS_abc**	48	TIGR01271	99.8	1.40E-24
**T1SS_abc**	49	TIGR03415	99.8	2.50E-24
**T1SS_abc**	50	TIGR02323	99.7	1.80E-23
**T1SS_abc**	51	TIGR03873	99.7	3.60E-23
**T1SS_abc**	52	TIGR03411	99.7	4.70E-23
**T1SS_abc**	53	TIGR03269	99.7	1.00E-22
**T1SS_abc**	54	TIGR01194	99.7	2.50E-22
**T1SS_abc**	55	TIGR02770	99.7	1.50E-21
**T1SS_abc**	56	TIGR02633	99.6	2.40E-21
**T1SS_abc**	57	TIGR00954	99.6	4.10E-21
**T1SS_abc**	58	TIGR03269	99.6	1.30E-20
**T1SS_abc**	59	TIGR03719	99.6	1.80E-20
**T1SS_abc**	60	TIGR01978	99.5	1.70E-19
**T1SS_abc**	61	TIGR00956	99.4	4.40E-18
**T1SS_abc**	62	TIGR01257	99.2	2.90E-16
**T1SS_abc**	63	TIGR02633	99	2.60E-14
**T1SS_abc**	64	TIGR01257	98.9	1.40E-13
**T1SS_abc**	65	TIGR00956	98.6	6.20E-12
**T1SS_abc**	66	TIGR00630	98.5	6.60E-12
**T1SS_mfp**	1	TIGR01843	100	1.20E-88
**T1SS_mfp**	2	TIGR00998	99.7	4.80E-23
**T1SS_mfp**	3	TIGR03794	99.5	3.70E-19
**T1SS_mfp**	4	TIGR01730	99.4	7.60E-18
**T1SS_mfp**	5	TIGR02971	99.2	7.20E-16
**T1SS_mfp**	6	TIGR01000	98.9	1.10E-13
**T1SS_mfp**	7	TIGR00999	98.5	9.20E-12
**T1SS_omf**	1	TIGR01844	100	1.40E-93
**T1SS_omf**	2	TIGR01845	100	2.40E-37
**T1SS_omf**	3	TIGR01844	99.5	1.50E-18
**T1SS_omf**	4	TIGR01845	98.5	1.80E-11
**T2SS_gspC**	1	TIGR01713	98.5	1.30E-11
**T2SS_gspD**	1	TIGR02517	100	4.00E-179
**T2SS_gspD**	2	TIGR02515	100	6.50E-54
**T2SS_gspD**	3	TIGR02516	100	8.30E-39
**T2SS_gspD**	4	TIGR02519	100	4.60E-37
**T2SS_gspD**	5	TIGR02517	99	9.80E-15
**T2SS_gspE**	1	TIGR02533	100	1.00E-188
**T2SS_gspE**	2	TIGR02538	100	6.00E-130
**T2SS_gspE**	3	TIGR01420	100	2.40E-54
**T2SS_gspE**	4	TIGR02525	99.9	9.00E-28
**T2SS_gspE**	5	TIGR02524	99.5	1.90E-19
**T2SS_gspE**	6	TIGR02782	98.9	1.50E-13
**T2SS_gspE**	7	TIGR02788	98.7	1.00E-12

**T2SS_gspE**	8	TIGR03819	98.1	3.40E-10
**T2SS_gspF**	1	TIGR02120	100	5.00E-163
**T2SS_gspF**	2	TIGR02120	99.1	8.20E-15
**T2SS_gspG**	1	TIGR01710	100	3.10E-71
**T2SS_gspH**	1	TIGR01708	100	5.60E-41
**T2SS_gspI**	1	TIGR01707	100	1.40E-43
**T2SS_gspJ**	1	TIGR01711	100	5.20E-37
**T2SS_gspL**	1	TIGR01709	100	6.60E-41
**T3SS_sctC**	1	TIGR02516	100	7.20E-87
**T3SS_sctC**	2	TIGR02517	100	1.70E-60
**T3SS_sctC**	3	TIGR02515	100	2.00E-38
**T3SS_sctC**	4	TIGR02519	99.9	1.90E-28
**T3SS_sctJ**	1	TIGR02544	100	6.90E-75
**T3SS_sctJ**	2	TIGR00206	99	9.60E-15
**T3SS_sctN**	1	TIGR02546	100	6.00E-198
**T3SS_sctN**	2	TIGR01026	100	7.00E-170
**T3SS_sctN**	3	TIGR03497	100	9.00E-170
**T3SS_sctN**	4	TIGR03496	100	2.00E-160
**T3SS_sctN**	5	TIGR03498	100	6.00E-144
**T3SS_sctN**	6	TIGR03305	100	1.40E-46
**T3SS_sctN**	7	TIGR03324	100	5.10E-45
**T3SS_sctN**	8	TIGR01039	100	6.90E-45
**T3SS_sctN**	9	TIGR01041	100	6.20E-43
**T3SS_sctN**	10	TIGR00962	100	9.40E-43
**T3SS_sctN**	11	TIGR01043	100	2.40E-41
**T3SS_sctN**	12	TIGR01042	99.9	2.00E-32
**T3SS_sctN**	13	TIGR01040	99.9	1.70E-29
**T3SS_sctN**	14	TIGR00767	99.7	8.10E-23
**T3SS_sctQ**	1	TIGR02551	99.9	1.30E-27
**T3SS_sctQ**	2	TIGR02480	99.5	8.50E-19
**T3SS_sctR**	1	TIGR01102	100	2.00E-102
**T3SS_sctR**	2	TIGR01103	100	4.30E-62
**T3SS_sctS**	1	TIGR01403	100	9.60E-44
**T3SS_sctS**	2	TIGR01402	99.8	2.00E-25
**T3SS_sctT**	1	TIGR01401	100	6.00E-79
**T3SS_sctT**	2	TIGR01400	99.9	9.20E-33
**T3SS_sctU**	1	TIGR01404	100	5.00E-137
**T3SS_sctU**	2	TIGR00328	100	1.60E-90
**T3SS_sctU**	3	TIGR00789	99	4.10E-14
**T3SS_sctV**	1	TIGR01399	100	9.00E-227
**T3SS_sctV**	2	TIGR01398	100	3.00E-151
**T4P_pilAE**	1	TIGR01710	98.9	7.90E-14
**T4P_pilAE**	2	TIGR02532	98.5	6.60E-12
**T4P_pilAE**	3	TIGR01708	98.4	4.40E-11
**T4P_pilAE**	4	TIGR02596	98.1	3.40E-10
**T4P_pilB**	1	TIGR02538	100	1.00E-201

**T4P_pilB**	2	TIGR02533	100	2.00E-159
**T4P_pilB**	3	TIGR01420	100	3.80E-61
**T4P_pilB**	4	TIGR02525	99.8	7.40E-27
**T4P_pilB**	5	TIGR02524	99.5	5.60E-19
**T4P_pilB**	6	TIGR02782	98.9	1.50E-13
**T4P_pilB**	7	TIGR02788	98.6	5.80E-12
**T4P_pilC**	1	TIGR02120	100	7.40E-86
**T4P_pilC**	2	TIGR02120	99	1.00E-14
**T4P_pilI_pilV**	1	TIGR02523	100	9.80E-51
**T4P_pilM**	1	TIGR01175	100	1.10E-98
**T4P_pilM**	2	TIGR01174	99.6	8.50E-20
**T4P_pilM**	3	TIGR02529	98.3	9.00E-11
**T4P_pilQ**	1	TIGR02515	100	3.60E-84
**T4P_pilQ**	2	TIGR02517	100	5.00E-50
**T4P_pilQ**	3	TIGR02516	100	1.30E-37
**T4P_pilQ**	4	TIGR02519	100	4.10E-37
**T4P_pilQ**	5	TIGR02520	98.9	1.60E-13
**T4P_pilT_pilU**	1	TIGR01420	100	2.00E-163
**T4P_pilT_pilU**	2	TIGR02525	100	3.70E-52
**T4P_pilT_pilU**	3	TIGR02538	100	1.00E-51
**T4P_pilT_pilU**	4	TIGR02533	100	1.80E-51
**T4P_pilT_pilU**	5	TIGR02524	100	1.80E-46
**T4P_pilT_pilU**	6	TIGR02782	99.1	3.70E-15
**T4P_pilT_pilU**	7	TIGR02788	99.1	5.00E-15
**T4P_pilT_pilU**	8	TIGR03819	98.7	2.00E-12
**T4SS_B_traJ**	1	TIGR03782	100	5.00E-188
**T4SS_B_traK**	1	TIGR03781	100	7.00E-120
**T4SS_B_traM**	1	TIGR03779	100	3.00E-160
**T4SS_B_traN**	1	TIGR03780	100	5.00E-130
**T4SS_B_traP**	1	TIGR01391	99.3	5.30E-17
**T4SS_F_traE**	1	TIGR02761	100	5.00E-43
**T4SS_F_traF**	1	TIGR02740	100	3.60E-88
**T4SS_F_traF**	2	TIGR02739	100	9.90E-46
**T4SS_F_traF**	3	TIGR02738	99.7	6.20E-23
**T4SS_F_traK**	1	TIGR02756	100	1.50E-50
**T4SS_F_traL**	1	TIGR02762	100	1.30E-35
**T4SS_F_traN**	1	TIGR02750	100	1.50E-83
**T4SS_F_traV**	1	TIGR02747	99.9	6.40E-33
**T4SS_F_traW**	1	TIGR02743	100	4.00E-70
**T4SS_F_trbC**	1	TIGR02742	100	2.30E-51
**T4SS_G_tfc10**	1	TIGR03745	100	5.60E-58
**T4SS_G_tfc11**	1	TIGR03750	100	1.30E-52
**T4SS_G_tfc12**	1	TIGR03746	100	7.00E-113
**T4SS_G_tfc13**	1	TIGR03749	100	3.00E-111
**T4SS_G_tfc14**	1	TIGR03752	100	2.00E-163
**T4SS_G_tfc15**	1	TIGR03751	100	2.80E-66

**T4SS_G_tfc2**	1	TIGR03748	100	1.30E-51
**T4SS_G_tfc22**	1	TIGR03755	100	6.00E-176
**T4SS_G_tfc23**	1	TIGR03756	100	3.00E-139
**T4SS_G_tfc24**	1	TIGR03757	100	2.40E-58
**T4SS_G_tfc3**	1	TIGR03759	100	2.00E-101
**T4SS_G_tfc5**	1	TIGR03765	100	3.50E-54
**T4SS_G_tfc7**	1	TIGR03747	100	4.60E-99
**T4SS_G_tfc8**	1	TIGR01690	100	1.10E-42
**T4SS_G_tfc9**	1	TIGR03758	100	5.40E-39
**T4SS_I_traY**	1	TIGR04346	100	6.00E-105
**T4SS_MOBF**	1	TIGR02760	100	4.00E-123
**T4SS_MOBF**	2	TIGR02768	100	3.20E-72
**T4SS_MOBF**	3	TIGR02686	100	8.80E-64
**T4SS_MOBF**	4	TIGR02760	99.9	3.20E-32
**T4SS_MOBF**	5	TIGR01448	99.9	1.10E-31
**T4SS_MOBF**	6	TIGR01447	99.9	4.60E-30
**T4SS_MOBH**	1	TIGR03760	100	5.00E-40
**T4SS_MOBQ**	1	TIGR02768	100	2.00E-70
**T4SS_T_virB11**	1	TIGR02782	100	3.00E-138
**T4SS_T_virB11**	2	TIGR02788	100	1.10E-58
**T4SS_T_virB11**	3	TIGR03819	100	1.50E-50
**T4SS_T_virB11**	4	TIGR01420	98.8	4.70E-13
**T4SS_T_virB11**	5	TIGR02533	98.5	8.50E-12
**T4SS_T_virB11**	6	TIGR02538	98.1	6.30E-10
**T4SS_T_virB5**	1	TIGR02780	99.9	4.70E-30
**T4SS_T_virB5**	2	TIGR02791	99.3	1.20E-16
**T4SS_T_virB6**	1	TIGR02783	99.9	5.80E-33
**T4SS_T_virB9**	1	TIGR02775	100	9.40E-62
**T4SS_T_virB9**	2	TIGR02781	100	2.30E-57
**T4SS_t4cp1**	1	TIGR03743	99.3	4.60E-17
**T4SS_t4cp1**	2	TIGR02759	99.2	1.00E-15
**T4SS_t4cp1**	3	TIGR03754	98.7	1.40E-12
**T4SS_t4cp1**	4	TIGR02767	98.5	8.40E-12
**T4SS_t4cp2**	1	TIGR02759	99.9	3.10E-31
**T4SS_t4cp2**	2	TIGR03743	99.9	1.50E-28
**T4SS_t4cp2**	3	TIGR02767	99.8	1.10E-26
**T4SS_t4cp2**	4	TIGR03754	99.7	1.30E-21
**T4SS_traU**	1	TIGR02746	99.7	1.40E-21
**T4SS_traU**	2	TIGR00929	99.3	6.10E-17
**T4SS_virb4**	1	TIGR00929	100	2.60E-87
**T4SS_virb4**	2	TIGR02746	100	8.50E-80
**T4SS_virb4**	3	TIGR03744	100	7.20E-46
**T4SS_virb4**	4	TIGR03783	100	4.40E-37
**T4SS_virb4**	5	TIGR02759	98.7	1.50E-12
**T5aSS_PF03797**	1	TIGR01414	99.9	3.20E-33
**T5bSS_t5bss-tranlocator**	1	TIGR03303	98.9	1.20E-13

**T6SS_tssA**	1	TIGR03362	100	3.90E-80
**T6SS_tssA**	2	TIGR03363	100	6.00E-40
**T6SS_tssB**	1	TIGR03358	100	4.80E-67
**T6SS_tssC**	1	TIGR03355	100	3.00E-226
**T6SS_tssD**	1	TIGR03344	100	1.10E-56
**T6SS_tssE**	1	TIGR03357	100	2.10E-45
**T6SS_tssF**	1	TIGR03359	100	2.00E-191
**T6SS_tssG**	1	TIGR03347	100	1.00E-102
**T6SS_tssH**	1	TIGR03345	100	0.00E+00
**T6SS_tssH**	2	TIGR03346	100	7.00E-205
**T6SS_tssH**	3	TIGR02639	100	5.00E-164
**T6SS_tssH**	4	TIGR00382	98.2	1.30E-10
**T6SS_tssI**	1	TIGR03361	100	1.00E-178
**T6SS_tssI**	2	TIGR01646	100	3.00E-145
**T6SS_tssJ**	1	TIGR03352	100	7.70E-49
**T6SS_tssK**	1	TIGR03353	100	4.00E-126
**T6SS_tssL**	1	TIGR03349	100	6.60E-66
**T6SS_tssM**	1	TIGR03348	100	2.00E-241
**T6SSii_iglA**	1	TIGR03358	100	3.20E-45
**T6SSii_iglB**	1	TIGR03355	100	1.00E-233
**T6SSiii_tssH**	1	TIGR03346	100	3.00E-161
**T6SSiii_tssH**	2	TIGR02639	100	8.00E-136
**T6SSiii_tssH**	3	TIGR03345	100	7.00E-124
**T6SSiii_tssH**	4	TIGR00382	98.1	3.10E-10
**T6SSiii_tssI**	1	TIGR03361	100	9.20E-36
**T6SSiii_tssI**	2	TIGR01646	99.9	3.00E-33
**T9SS_sprA_PF14349**	1	TIGR04189	100	3.00E-158
**T9SS_sprA_PF14349**	2	TIGR04189	100	2.00E-34
**Tad_rcpA**	1	TIGR02517	99.9	2.80E-33
**Tad_rcpA**	2	TIGR02515	99.9	2.70E-28
**Tad_rcpA**	3	TIGR02519	99.8	5.70E-24
**Tad_rcpA**	4	TIGR02516	99.5	1.30E-19
**Tad_tadA**	1	TIGR03819	100	6.40E-93
**Tad_tadA**	2	TIGR02788	100	2.40E-68
**Tad_tadA**	3	TIGR02782	100	4.40E-59
**Tad_tadA**	4	TIGR02525	99.1	8.30E-15
**Tad_tadA**	5	TIGR01420	99	1.80E-14
**Tad_tadA**	6	TIGR02533	98.7	9.40E-13
**Tad_tadA**	7	TIGR02538	98.6	2.50E-12
**Tad_tadZ**	1	TIGR01968	98.9	5.00E-14
**Tad_tadZ**	2	TIGR03815	98.8	4.50E-13
**Tad_tadZ**	3	TIGR01969	98.6	2.70E-12
**Tad_tadZ**	4	TIGR03371	98.6	4.40E-12

Table S7. Comparison of the number of predicted T3SS and T6SS^{i} between TXSScan and T346Hunter.

See the file TableS7.xls attached to the manuscript.

Text S1. Comparison of TXSScan predictions of T3SS and T6SS with T346Hunter's predictions

TXSScan can be run on any custom data with the program MacSyFinder. Most existing methods are made available on webservers (Table 1) and most of the time, the predictions cannot be downloaded in block.

Nevertheless, we compared our predictions to that of T346Hunter. As we could not retrieve the details of the systems themselves (only the genes with hits for profiles can be retrieved from the website, not the genes in predicted clusters), we could only compare the number of predicted systems in each replicon. We downloaded the prediction results from the T346Hunter's paper ([124], Table S2). We compared these systems with our predicted systems stored in Table S3.

We could perform such a comparison for the T3SS and the T6SSi predicted in 1440 replicons. We propose specific models and profiles for two types of T6SS, T6SSii and T6SSiii, which are not covered by T346Hunter. Profiles used in T346Hunter are based on the homologs of the 1st T6SS loci described, i.e., the T6SSi from *V. cholerae, P. aeruginosa and B. mallei*, see [125]). We therefore did not include our predictions for these systems in the comparison. In the case of the T4SS, T346Hunter did not provide the information on the type of system predicted: conjugative, or potentially involved in protein secretion. It therefore impeded a fair comparison.

T346Hunter predicts putative loci of systems as soon as four "core components" of the system are found contiguous in the genomes, whatever the system. It does not attempt to predict complete systems. Our models propose a "quorum" tailored for each system, as our aim is to predict putatively functional systems (7/9 core components for the T3SS and 11/14

for the T6SS). It means that the two tools do not aim to predict exactly the same thing. The results are presented in Table S7.

Briefly, T346Hunter and TXSScan agreed on the number of predicted systems for 1401 out of 1440 replicons (i.e., 97.5%) for the T3SS, and for 1314 out of 1440 replicons (91.2%) for the T6SS. As expected from the criteria used by T346Hunter and by our models, we are much more stringent in our prediction, and predict less systems in most of the cases (39 and 126 cases for T3SS and T6SS resp.). Some of the systems not predicted by TXSScan corresponded to partial or degraded systems (e.g., one of the T3SS of *E. coli*, ETT2 [126], and the T6SS of *Acinetobacter baumannii* ATCC 17978, never shown to be involved in virulence or bacterial competition, [107,127]), or variants of systems that may not be working as protein secretion systems (case of the T3SS predicted for Myxococcales, an evolutionary intermediate between Flagellum and T3SS, see our paper from 2012, [41]). In some cases, TXSScan predicted systems from scattered loci ("multi-loci" systems), when T346Hunter predicts one system for each locus, for example for the predicted T6SS in *Acidiphilium cryptum* JF 5. The fact that T6SS putative components tend to be more scattered in the genomes than T3SS components (see main text) explain partly the larger number of discrepancies for the T6SS. An extreme case was the one of *Photorhabdus asymbiotica*, where six loci were predicted by T346Hunter, and three systems inferred by TXSScan. The "correct answer" seems to lie in between, but is not totally clear, as it seems that this genome has four loci with T6SS components, one looking like two contiguous systems. In the latest case, TXSScan counts only one system as it does not "cut" into predicted systems that co-localize according to the defined co-localization criterion, whereas T346Hunter counts three systems, with respectively 12, 5, and 7 of the core components. In addition, TXSScan misses one of this strain's system because it does not pass the core component threshold (10 of the 11 "mandatory" components).

To summarize, there are few differences between the two methods for the T3SS and T6SSi. Most differences resulted from 1) a difference in the requirements (mostly the quorum, but also the co-localization criteria) to select a cluster as part of a putative system, which

increases the predictions of T346Hunter and decreases that of TXSScan; 2) the fact that TXSScan is able to predict putative complete systems scattered in several loci.

File S1. Models and profiles for the detection of protein secretion systems and related appendages with MacSyFinder.

The archive is available at:

https://researchpullzone-yhello.netdna-ssl.com/wp-content/uploads/2015/10/research.pasteur.fr_txsscan-models-and-profiles-for-protein-secretion-systems-1.gz

References

1 Guglielmini, J., de la Cruz, F. & Rocha, E. P. Evolution of conjugation and type IV secretion systems. *Mol. Biol. Evol.* **30**, 315-331, doi:10.1093/molbev/mss221 (2013).

2 Delepelaire, P. Type I secretion in gram-negative bacteria. *Biochim. Biophys. Acta* **1694**, 149-161, doi:10.1016/j.bbamcr.2004.05.001 (2004).

3 Bouige, P., Laurent, D., Piloyan, L. & Dassa, E. Phylogenetic and functional classification of ATP-binding cassette (ABC) systems. *Curr. Protein Pept. Sci.* **3**, 541-559 (2002).

4 Dassa, E. & Bouige, P. The ABC of ABCS: a phylogenetic and functional classification of ABC systems in living organisms. *Res. Microbiol.* **152**, 211-229 (2001).

5 Gilson, L., Mahanty, H. K. & Kolter, R. Genetic analysis of an MDR-like export system: the secretion of colicin V. *EMBO J.* **9**, 3875-3884 (1990).

6 Hess, J., Wels, W., Vogel, M. & Goebel, W. Nucleotide-Sequence of a Plasmid-Encoded Hemolysin Determinant and Its Comparison with a Corresponding Chromosomal Hemolysin Sequence. *FEMS Microbiol. Lett.* **34**, 1-11, doi:Doi 10.1016/0378-1097(86)90261-2 (1986).

7 Delepelaire, P. & Wandersman, C. Characterization, localization and transmembrane organization of the three proteins PrtD, PrtE and PrtF necessary for protease secretion by the gram-negative bacterium Erwinia chrysanthemi. *Mol. Microbiol.* **5**, 2427-2434 (1991).

8 Glaser, P., Sakamoto, H., Bellalou, J., Ullmann, A. & Danchin, A. Secretion of cyclolysin, the calmodulin-sensitive adenylate cyclase-haemolysin bifunctional protein of Bordetella pertussis. *EMBO J.* **7**, 3997-4004 (1988).

9 Binet, R. & Wandersman, C. Cloning of the Serratia marcescens hasF gene encoding the Has ABC exporter outer membrane component: a TolC analogue. *Mol. Microbiol.* **22**, 265-273 (1996).

10 Letoffe, S., Ghigo, J. M. & Wandersman, C. Secretion of the Serratia marcescens HasA protein by an ABC transporter. *J. Bacteriol.* **176**, 5372-5377 (1994).

11 Letoffe, S., Ghigo, J. M. & Wandersman, C. Iron acquisition from heme and hemoglobin by a Serratia marcescens extracellular protein. *Proc. Natl. Acad. Sci. U. S. A.* **91**, 9876-9880 (1994).

12 Akatsuka, H., Kawai, E., Omori, K. & Shibatani, T. The three genes lipB, lipC, and lipD involved in the extracellular secretion of the Serratia marcescens lipase which lacks an N-terminal signal peptide. *J. Bacteriol.* **177**, 6381-6389 (1995).

13 Finnie, C., Hartley, N. M., Findlay, K. C. & Downie, J. A. The Rhizobium leguminosarum prsDE genes are required for secretion of several proteins, some of which influence nodulation, symbiotic nitrogen fixation and exopolysaccharide modification. *Mol. Microbiol.* **25**, 135-146 (1997).

14 Awram, P. & Smit, J. The Caulobacter crescentus paracrystalline S-layer protein is secreted by an ABC transporter (type I) secretion apparatus. *J. Bacteriol.* **180**, 3062-3069 (1998).

15 Cianciotto, N. P. Type II secretion: a protein secretion system for all seasons. *Trends Microbiol.* **13**, 581-588, doi:10.1016/j.tim.2005.09.005 (2005).

16 Mehta, T., Childers, S. E., Glaven, R., Lovley, D. R. & Mester, T. A putative multicopper protein secreted by an atypical type II secretion system involved in the reduction of insoluble electron acceptors in Geobacter sulfurreducens. *Microbiology* **152**, 2257-2264, doi:10.1099/mic.0.28864-0 (2006).

17 DeShazer, D., Brett, P. J., Burtnick, M. N. & Woods, D. E. Molecular characterization of genetic loci required for secretion of exoproducts in Burkholderia pseudomallei. *J. Bacteriol.* **181**, 4661-4664 (1999).

18 Le Blastier, S. *et al.* Phosphate starvation triggers production and secretion of an extracellular lipoprotein in Caulobacter crescentus. *PLoS ONE* **5**, e14198, doi:10.1371/journal.pone.0014198 (2010).

19 Francetic, O. & Pugsley, A. P. The cryptic general secretory pathway (gsp) operon of Escherichia coli K-12 encodes functional proteins. *J. Bacteriol.* **178**, 3544-3549 (1996).

20 Durand, E. *et al.* The assembly mode of the pseudopilus: a hallmark to distinguish a novel secretion system subtype. *J. Biol. Chem.* **286**, 24407-24416, doi:10.1074/jbc.M111.234278 (2011).

21 Denny, T. P., Carney, B. F. & Schell, M. A. Inactivation of Multiple Virulence Genes Reduces the Ability of Pseudomonas-Solanacearum to Cause Wilt Symptoms. *Mol. Plant. Microbe Interact.* **3**, 293-300, doi:Doi 10.1094/Mpmi-3-293 (1990).

22 Hu, N. T. *et al.* Cloning and characterization of a gene required for the secretion of extracellular enzymes across the outer membrane by Xanthomonas campestris pv. campestris. *J. Bacteriol.* **174**, 2679-2687 (1992).
23 Lee, H. M. *et al.* Association of the cytoplasmic membrane protein XpsN with the outer membrane protein XpsD in the type II protein secretion apparatus of Xanthomonas campestris pv. campestris. *J. Bacteriol.* **182**, 1549-1557 (2000).
24 Pelicic, V. Type IV pili: e pluribus unum? *Mol. Microbiol.* **68**, 827-837, doi:10.1111/j.1365-2958.2008.06197.x (2008).
25 Bakaletz, L. O. *et al.* Demonstration of Type IV pilus expression and a twitching phenotype by Haemophilus influenzae. *Infect. Immun.* **73**, 1635-1643, doi:10.1128/IAI.73.3.1635-1643.2005 (2005).
26 Forslund, A. L. *et al.* Direct repeat-mediated deletion of a type IV pilin gene results in major virulence attenuation of Francisella tularensis. *Mol. Microbiol.* **59**, 1818-1830, doi:10.1111/j.1365-2958.2006.05061.x (2006).
27 Wu, S. S. & Kaiser, D. Genetic and functional evidence that Type IV pili are required for social gliding motility in Myxococcus xanthus. *Mol. Microbiol.* **18**, 547-558 (1995).
28 Tonjum, T. & Koomey, M. The pilus colonization factor of pathogenic neisserial species: organelle biogenesis and structure/function relationships--a review. *Gene* **192**, 155-163 (1997).
29 Duggan, P. S., Gottardello, P. & Adams, D. G. Molecular analysis of genes in Nostoc punctiforme involved in pilus biogenesis and plant infection. *J. Bacteriol.* **189**, 4547-4551, doi:10.1128/JB.01927-06 (2007).
30 Alm, R. A. & Mattick, J. S. Genes involved in the biogenesis and function of type-4 fimbriae in Pseudomonas aeruginosa. *Gene* **192**, 89-98 (1997).
31 Liu, H., Kang, Y., Genin, S., Schell, M. A. & Denny, T. P. Twitching motility of Ralstonia solanacearum requires a type IV pilus system. *Microbiology* **147**, 3215-3229 (2001).
32 Friedrich, A., Rumszauer, J., Henne, A. & Averhoff, B. Pilin-like proteins in the extremely thermophilic bacterium Thermus thermophilus HB27: implication in competence for natural transformation and links to type IV pilus biogenesis. *Appl. Environ. Microbiol.* **69**, 3695-3700 (2003).
33 Tomich, M., Planet, P. J. & Figurski, D. H. The tad locus: postcards from the widespread colonization island. *Nat. Rev. Microbiol.* **5**, 363-375 (2007).
34 Haase, E. M., Zmuda, J. L. & Scannapieco, F. A. Identification and molecular analysis of rough-colony-specific outer membrane proteins of Actinobacillus actinomycetemcomitans. *Infect. Immun.* **67**, 2901-2908 (1999).
35 Kachlany, S. C. *et al.* Nonspecific adherence by Actinobacillus actinomycetemcomitans requires genes widespread in bacteria and archaea. *J. Bacteriol.* **182**, 6169-6176 (2000).
36 Skerker, J. M. & Shapiro, L. Identification and cell cycle control of a novel pilus system in Caulobacter crescentus. *EMBO J.* **19**, 3223-3234, doi:10.1093/emboj/19.13.3223 (2000).
37 Nika, J. R. *et al.* Haemophilus ducreyi requires the flp gene cluster for microcolony formation in vitro. *Infect. Immun.* **70**, 2965-2975 (2002).
38 Bernard, C. S., Bordi, C., Termine, E., Filloux, A. & de Bentzmann, S. Organization and PprB-dependent control of the Pseudomonas aeruginosa tad Locus, involved in Flp pilus biology. *J. Bacteriol.* **191**, 1961-1973, doi:10.1128/JB.01330-08 (2009).
39 Wairuri, C. K., van der Waals, J. E., van Schalkwyk, A. & Theron, J. Ralstonia solanacearum needs Flp pili for virulence on potato. *Mol. Plant. Microbe Interact.* **25**, 546-556, doi:10.1094/MPMI-06-11-0166 (2012).
40 Schilling, J. *et al.* Transcriptional activation of the tad type IVb pilus operon by PypB in Yersinia enterocolitica. *J. Bacteriol.* **192**, 3809-3821 (2010).
41 Abby, S. S. & Rocha, E. P. The non-flagellar type III secretion system evolved from the bacterial flagellum and diversified into host-cell adapted systems. *PLoS Genet.* **8**, e1002983, doi:10.1371/journal.pgen.1002983 (2012).
42 Guglielmini, J., Quintais, L., Garcillan-Barcia, M. P., de la Cruz, F. & Rocha, E. P. The Repertoire of ICE in Prokaryotes Underscores the Unity, Diversity, and Ubiquity of Conjugation. *PLoS Genet.* **7**, e1002222, doi:10.1371/journal.pgen.1002222
PGENETICS-D-11-00532 [pii] (2011).
43 Dautin, N. & Bernstein, H. D. Protein Secretion in Gram-Negative Bacteria via the Autotransporter Pathway. *Annu. Rev. Microbiol.* **61**, 89-112, doi:10.1146/annurev.micro.61.080706.093233 (2007).

44 Yen, M. R. *et al.* Protein-translocating outer membrane porins of Gram-negative bacteria. *Biochim. Biophys. Acta* **1562**, 6-31 (2002).
45 Ward, C. K., Mock, J. R. & Hansen, E. J. The LspB protein is involved in the secretion of the LspA1 and LspA2 proteins by Haemophilus ducreyi. *Infect. Immun.* **72**, 1874-1884 (2004).
46 Hirono, I., Tange, N. & Aoki, T. Iron-regulated haemolysin gene from Edwardsiella tarda. *Mol. Microbiol.* **24**, 851-856 (1997).
47 Strauss, E. J., Ghori, N. & Falkow, S. An Edwardsiella tarda strain containing a mutation in a gene with homology to shlB and hpmB is defective for entry into epithelial cells in culture. *Infect. Immun.* **65**, 3924-3932 (1997).
48 Kida, Y., Higashimoto, Y., Inoue, H., Shimizu, T. & Kuwano, K. A novel secreted protease from Pseudomonas aeruginosa activates NF-kappaB through protease-activated receptors. *Cell. Microbiol.* **10**, 1491-1504, doi:10.1111/j.1462-5822.2008.01142.x (2008).
49 Molina, M. A., Ramos, J. L. & Espinosa-Urgel, M. A two-partner secretion system is involved in seed and root colonization and iron uptake by Pseudomonas putida KT2440. *Environ. Microbiol.* **8**, 639-647, doi:10.1111/j.1462-2920.2005.00940.x (2006).
50 Hodak, H. *et al.* Secretion signal of the filamentous haemagglutinin, a model two-partner secretion substrate. *Mol. Microbiol.* **61**, 368-382, doi:10.1111/j.1365-2958.2006.05242.x (2006).
51 Poole, K., Schiebel, E. & Braun, V. Molecular characterization of the hemolysin determinant of Serratia marcescens. *J. Bacteriol.* **170**, 3177-3188 (1988).
52 Boyer, F., Fichant, G., Berthod, J., Vandenbrouck, Y. & Attree, I. Dissecting the bacterial type VI secretion system by a genome wide in silico analysis: what can be learned from available microbial genomic resources? *BMC Genomics* **10**, 104, doi:1471-2164-10-104 [pii]
10.1186/1471-2164-10-104 (2009).
53 Suarez, G. *et al.* Molecular characterization of a functional type VI secretion system from a clinical isolate of Aeromonas hydrophila. *Microb. Pathog.* **44**, 344-361 (2008).
54 Schell, M. A. *et al.* Type VI secretion is a major virulence determinant in Burkholderia mallei. *Mol. Microbiol.* **64**, 1466-1485, doi:MMI5734 [pii]
10.1111/j.1365-2958.2007.05734.x (2007).
55 Shalom, G., Shaw, J. G. & Thomas, M. S. In vivo expression technology identifies a type VI secretion system locus in Burkholderia pseudomallei that is induced upon invasion of macrophages. *Microbiology* **153**, 2689-2699, doi:10.1099/mic.0.2007/006585-0 (2007).
56 Mougous, J. D. *et al.* A virulence locus of Pseudomonas aeruginosa encodes a protein secretion apparatus. *Science* **312**, 1526-1530, doi:312/5779/1526 [pii]
10.1126/science.1128393 (2006).
57 Records, A. R. & Gross, D. C. Sensor kinases RetS and LadS regulate Pseudomonas syringae type VI secretion and virulence factors. *J. Bacteriol.* **192**, 3584-3596, doi:10.1128/JB.00114-10 (2010).
58 Pukatzki, S. *et al.* Identification of a conserved bacterial protein secretion system in Vibrio cholerae using the Dictyostelium host model system. *Proc. Natl. Acad. Sci. U. S. A.* **103**, 1528-1533, doi:10.1073/pnas.0510322103 (2006).
59 Lloyd, A. L., Henderson, T. A., Vigil, P. D. & Mobley, H. L. Genomic islands of uropathogenic Escherichia coli contribute to virulence. *J. Bacteriol.* **191**, 3469-3481, doi:10.1128/JB.01717-08 (2009).
60 Wang, L. *et al.* Cell density- and quorum sensing-dependent expression of type VI secretion system 2 in Vibrio parahaemolyticus. *PLoS ONE* **8**, e73363, doi:10.1371/journal.pone.0073363 (2013).
61 Nano, F. E. *et al.* A Francisella tularensis pathogenicity island required for intramacrophage growth. *J. Bacteriol.* **186**, 6430-6436, doi:10.1128/JB.186.19.6430-6436.2004 (2004).
62 Broms, J. E., Sjostedt, A. & Lavander, M. The Role of the Francisella Tularensis Pathogenicity Island in Type VI Secretion, Intracellular Survival, and Modulation of Host Cell Signaling. *Front. Microbiol.* **1**, 136, doi:10.3389/fmicb.2010.00136 (2010).
63 Russell, A. B. *et al.* A Type VI Secretion-Related Pathway in Bacteroidetes Mediates Interbacterial Antagonism. *Cell Host Microbe* **16**, 227-236, doi:10.1016/j.chom.2014.07.007 (2014).
64 Shrivastava, A., Johnston, J. J., van Baaren, J. M. & McBride, M. J. Flavobacterium johnsoniae GldK, GldL, GldM, and SprA are required for secretion of the cell surface gliding motility adhesins SprB and RemA. *J. Bacteriol.* **195**, 3201-3212, doi:10.1128/JB.00333-13 (2013).

65 Sato, K. *et al.* A protein secretion system linked to bacteroidete gliding motility and pathogenesis. *Proc. Natl. Acad. Sci. U. S. A.* **107**, 276-281, doi:10.1073/pnas.0912010107 (2010).
66 Bleves, S. *et al.* Protein secretion systems in Pseudomonas aeruginosa: A wealth of pathogenic weapons. *Int. J. Med. Microbiol.* **300**, 534-543, doi:10.1016/j.ijmm.2010.08.005 (2010).
67 Ma, Q., Zhai, Y., Schneider, J. C., Ramseier, T. M. & Saier, M. H., Jr. Protein secretion systems of Pseudomonas aeruginosa and P fluorescens. *Biochim. Biophys. Acta* **1611**, 223-233 (2003).
68 Shrivastava, R. & Miller, J. F. Virulence factor secretion and translocation by Bordetella species. *Curr. Opin. Microbiol.* **12**, 88-93 (2009).
69 Chenal, A., Guijarro, J. I., Raynal, B., Delepierre, M. & Ladant, D. RTX calcium binding motifs are intrinsically disordered in the absence of calcium: implication for protein secretion. *J. Biol. Chem.* **284**, 1781-1789, doi:10.1074/jbc.M807312200 (2009).
70 Gerlach, R. G. *et al.* Salmonella Pathogenicity Island 4 encodes a giant non-fimbrial adhesin and the cognate type 1 secretion system. *Cell. Microbiol.* **9**, 1834-1850, doi:10.1111/j.1462-5822.2007.00919.x (2007).
71 Binet, R., Letoffe, S., Ghigo, J. M., Delepelaire, P. & Wandersman, C. Protein secretion by Gram-negative bacterial ABC exporters--a review. *Gene* **192**, 7-11 (1997).
72 Burland, V. *et al.* The complete DNA sequence and analysis of the large virulence plasmid of Escherichia coli O157:H7. *Nucleic Acids Res.* **26**, 4196-4204 (1998).
73 Lathem, W. W. *et al.* StcE, a metalloprotease secreted by Escherichia coli O157:H7, specifically cleaves C1 esterase inhibitor. *Mol. Microbiol.* **45**, 277-288 (2002).
74 Tauschek, M., Gorrell, R. J., Strugnell, R. A. & Robins-Browne, R. M. Identification of a protein secretory pathway for the secretion of heat-labile enterotoxin by an enterotoxigenic strain of Escherichia coli. *Proc. Natl. Acad. Sci. U. S. A.* **99**, 7066-7071, doi:10.1073/pnas.092152899 (2002).
75 Baldi, D. L. *et al.* The type II secretion system and its ubiquitous lipoprotein substrate, SslE, are required for biofilm formation and virulence of enteropathogenic Escherichia coli. *Infect. Immun.* **80**, 2042-2052, doi:10.1128/IAI.06160-11 (2012).
76 Kornacker, M. G., Boyd, A., Pugsley, A. P. & Plastow, G. S. Klebsiella pneumoniae strain K21: evidence for the rapid secretion of an unacylated form of pullulanase. *Mol. Microbiol.* **3**, 497-503 (1989).
77 Pugsley, A. P. The complete general secretory pathway in gram-negative bacteria. *Microbiol. Mol. Biol. Rev.* **57**, 50-108 (1993).
78 Ferrandez, Y. & Condemine, G. Novel mechanism of outer membrane targeting of proteins in Gram-negative bacteria. *Mol. Microbiol.* **69**, 1349-1357, doi:10.1111/j.1365-2958.2008.06366.x (2008).
79 Sandkvist, M. Biology of type II secretion. *Mol. Microbiol.* **40**, 271-283 (2001).
80 Cianciotto, N. P. Many substrates and functions of type II secretion: lessons learned from Legionella pneumophila. *Future Microbiol.* **4**, 797-805, doi:10.2217/fmb.09.53 (2009).
81 Costa, J., d'Avo, A. F., da Costa, M. S. & Verissimo, A. Molecular evolution of key genes for type II secretion in Legionella pneumophila. *Environ. Microbiol.* **14**, 2017-2033, doi:10.1111/j.1462-2920.2011.02646.x (2012).
82 Iwobi, A. *et al.* Novel virulence-associated type II secretion system unique to high-pathogenicity Yersinia enterocolitica. *Infect. Immun.* **71**, 1872-1879 (2003).
83 Parrilli, E., Giuliani, M. & Tutino, M. L. General Secretory Pathway from marine Antarctic Pseudoalteromonas haloplanktis TAC125. *Mar Genomics* **1**, 123-128, doi:10.1016/j.margen.2009.01.002 (2008).
84 Sanchez-Porro, C., Mellado, E., Pugsley, A. P., Francetic, O. & Ventosa, A. The Haloprotease CPI Produced by the Moderately Halophilic Bacterium Pseudoalteromonas ruthenica Is Secreted by the Type II Secretion Pathway. *Appl. Environ. Microbiol.* **75**, 4197-4201, doi:Doi 10.1128/Aem.00156-09 (2009).
85 de la Haba, R. R., Sanchez-Porro, C., Leon, M. J., Papke, R. T. & Ventosa, A. Draft Genome Sequence of the Moderately Halophilic Bacterium Pseudoalteromonas ruthenica Strain CP76. *Genome Announc* **1**, doi:10.1128/genomeA.00268-13 (2013).
86 Shi, L. *et al.* Direct involvement of type II secretion system in extracellular translocation of Shewanella oneidensis outer membrane cytochromes MtrC and OmcA. *J. Bacteriol.* **190**, 5512-5516, doi:Doi 10.1128/Jb.00514-08 (2008).

87 Connell, T. D., Metzger, D. J., Lynch, J. & Folster, J. P. Endochitinase is transported to the extracellular milieu by the eps-encoded general secretory pathway of Vibrio cholerae. *J. Bacteriol.* **180**, 5591-5600 (1998).

88 Sandkvist, M. *et al.* General secretion pathway (eps) genes required for toxin secretion and outer membrane biogenesis in Vibrio cholerae. *J. Bacteriol.* **179**, 6994-7003 (1997).

89 Hwang, W. *et al.* Functional characterization of EpsC, a component of the type II secretion system, in the pathogenicity of Vibrio vulnificus. *Infect. Immun.* **79**, 4068-4080, doi:10.1128/IAI.05351-11 (2011).

90 Karaba, S. M., White, R. C. & Cianciotto, N. P. Stenotrophomonas maltophilia Encodes a Type II Protein Secretion System That Promotes Detrimental Effects on Lung Epithelial Cells. *Infect. Immun.* **81**, 3210-3219, doi:Doi 10.1128/Iai.00546-13 (2013).

91 Seitz, P. & Blokesch, M. DNA-uptake machinery of naturally competent Vibrio cholerae. *Proc. Natl. Acad. Sci. U. S. A.* **110**, 17987-17992, doi:10.1073/pnas.1315647110 (2013).

92 Bahar, O., Goffer, T. & Burdman, S. Type IV Pili are required for virulence, twitching motility, and biofilm formation of acidovorax avenae subsp. Citrulli. *Mol. Plant. Microbe Interact.* **22**, 909-920, doi:10.1094/MPMI-22-8-0909 (2009).

93 Dulla, G. F., Go, R. A., Stahl, D. A. & Davidson, S. K. Verminephrobacter eiseniae type IV pili and flagella are required to colonize earthworm nephridia. *ISME J.* **6**, 1166-1175, doi:10.1038/ismej.2011.183 (2012).

94 Boyd, J. M. *et al.* Contribution of type IV pili to the virulence of Aeromonas salmonicida subsp. salmonicida in Atlantic salmon (Salmo salar L.). *Infect. Immun.* **76**, 1445-1455, doi:10.1128/IAI.01019-07 (2008).

95 Xu, Q. *et al.* Structure of the pilus assembly protein TadZ from Eubacterium rectale: implications for polar localization. *Mol. Microbiol.* **83**, 712-727, doi:10.1111/j.1365-2958.2011.07954.x (2012).

96 Nykyri, J. *et al.* Role and Regulation of the Flp/Tad Pilus in the Virulence of Pectobacterium atrosepticum SCRI1043 and Pectobacterium wasabiae SCC3193. *PLoS ONE* **8**, e73718, doi:10.1371/journal.pone.0073718 (2013).

97 Luckett, J. C. *et al.* A novel virulence strategy for Pseudomonas aeruginosa mediated by an autotransporter with arginine-specific aminopeptidase activity. *PLoS Pathog.* **8**, e1002854, doi:10.1371/journal.ppat.1002854 (2012).

98 van der Woude, M. W. & Henderson, I. R. Regulation and function of Ag43 (flu). *Annu. Rev. Microbiol.* **62**, 153-169, doi:10.1146/annurev.micro.62.081307.162938 (2008).

99 Ulett, G. C. *et al.* Functional analysis of antigen 43 in uropathogenic Escherichia coli reveals a role in long-term persistence in the urinary tract. *Infect. Immun.* **75**, 3233-3244, doi:10.1128/IAI.01952-06 (2007).

100 Brennan, M. J. *et al.* Identification of a 69-kilodalton nonfimbrial protein as an agglutinogen of Bordetella pertussis. *Infect. Immun.* **56**, 3189-3195 (1988).

101 Aoki, S. K. *et al.* Contact-dependent inhibition of growth in Escherichia coli. *Science* **309**, 1245-1248, doi:10.1126/science.1115109 (2005).

102 Choi, P. S., Dawson, A. J. & Bernstein, H. D. Characterization of a novel two-partner secretion system in Escherichia coli O157:H7. *J. Bacteriol.* **189**, 3452-3461, doi:10.1128/JB.01751-06 (2007).

103 Ruer, S., Ball, G., Filloux, A. & de Bentzmann, S. The 'P-usher', a novel protein transporter involved in fimbrial assembly and TpsA secretion. *EMBO J.* **27**, 2669-2680, doi:10.1038/emboj.2008.197 (2008).

104 Totsika, M. *et al.* Molecular characterization of the EhaG and UpaG trimeric autotransporter proteins from pathogenic Escherichia coli. *Appl. Environ. Microbiol.* **78**, 2179-2189, doi:10.1128/AEM.06680-11 (2012).

105 Valle, J. *et al.* UpaG, a new member of the trimeric autotransporter family of adhesins in uropathogenic Escherichia coli. *J. Bacteriol.* **190**, 4147-4161, doi:10.1128/JB.00122-08 (2008).

106 Zheng, J. & Leung, K. Y. Dissection of a type VI secretion system in Edwardsiella tarda. *Mol. Microbiol.* **66**, 1192-1206, doi:10.1111/j.1365-2958.2007.05993.x (2007).

107 Weber, B. S. *et al.* Genomic and functional analysis of the type VI secretion system in Acinetobacter. *PLoS ONE* **8**, e55142, doi:10.1371/journal.pone.0055142 (2013).

108 Gueguen, E. & Cascales, E. Promoter swapping unveils the role of the Citrobacter rodentium CTS1 type VI secretion system in interbacterial competition. *Appl. Environ. Microbiol.* **79**, 32-38, doi:10.1128/AEM.02504-12 (2013).

109 Wu, C. F., Lin, J. S., Shaw, G. C. & Lai, E. M. Acid-induced type VI secretion system is regulated by ExoR-ChvG/ChvI signaling cascade in Agrobacterium tumefaciens. *PLoS Pathog.* **8**, e1002938, doi:10.1371/journal.ppat.1002938 (2012).

110 Aubert, D. F., Flannagan, R. S. & Valvano, M. A. A novel sensor kinase-response regulator hybrid controls biofilm formation and type VI secretion system activity in Burkholderia cenocepacia. *Infect. Immun.* **76**, 1979-1991, doi:10.1128/IAI.01338-07 (2008).

111 Pieper, R. *et al.* Temperature and growth phase influence the outer-membrane proteome and the expression of a type VI secretion system in Yersinia pestis. *Microbiology* **155**, 498-512 (2009).

112 Dudley, E. G., Thomson, N. R., Parkhill, J., Morin, N. P. & Nataro, J. P. Proteomic and microarray characterization of the AggR regulon identifies a pheU pathogenicity island in enteroaggregative Escherichia coli. *Mol. Microbiol.* **61**, 1267-1282, doi:10.1111/j.1365-2958.2006.05281.x (2006).

113 Guglielmini, J. *et al.* Key components of the eight classes of type IV secretion systems involved in bacterial conjugation or protein secretion. *Nucleic Acids Res.*, doi:10.1093/nar/gku194 (2014).

114 Edgar, R. C. MUSCLE: multiple sequence alignment with high accuracy and high throughput. *Nucleic Acids Res.* **32**, 1792-1797 (2004).

115 Gouy, M., Guindon, S. & Gascuel, O. SeaView version 4: A multiplatform graphical user interface for sequence alignment and phylogenetic tree building. *Mol. Biol. Evol.* **27**, 221-224, doi:msp259 [pii]
10.1093/molbev/msp259 (2010).

116 Enright, A. J., Van Dongen, S. & Ouzounis, C. A. An efficient algorithm for large-scale detection of protein families. *Nucleic Acids Res.* **30**, 1575-1584 (2002).

117 Miele, V., Penel, S. & Duret, L. Ultra-fast sequence clustering from similarity networks with SiLiX. *BMC Bioinformatics* **12**, 116 (2011).

118 Notredame, C., Higgins, D. G. & Heringa, J. T-Coffee: A novel method for fast and accurate multiple sequence alignment. *J. Mol. Biol.* **302**, 205-217, doi:10.1006/jmbi.2000.4042 (2000).

119 Katoh, K. & Toh, H. Recent developments in the MAFFT multiple sequence alignment program. *Brief. Bioinform.* **9**, 286-298, doi:10.1093/bib/bbn013 (2008).

120 Finn, R. D. *et al.* The Pfam protein families database. *Nucleic Acids Res.* **36**, D281-288 (2008).

121 Katoh, K. & Toh, H. Parallelization of the MAFFT multiple sequence alignment program. *Bioinformatics* **26**, 1899-1900, doi:btq224 [pii]
10.1093/bioinformatics/btq224 (2010).

122 Criscuolo, A. & Gribaldo, S. BMGE (Block Mapping and Gathering with Entropy): a new software for selection of phylogenetic informative regions from multiple sequence alignments. *BMC Evol. Biol.* **10**, 210 (2010).

123 Soding, J. Protein homology detection by HMM-HMM comparison. *Bioinformatics* **21**, 951-960, doi:10.1093/bioinformatics/bti125 (2005).

124 Martinez-Garcia, P. M., Ramos, C. & Rodriguez-Palenzuela, P. T346Hunter: a novel web-based tool for the prediction of type III, type IV and type VI secretion systems in bacterial genomes. *PLoS ONE* **10**, e0119317, doi:10.1371/journal.pone.0119317 (2015).

125 Shrivastava, S. & Mande, S. S. Identification and functional characterization of gene components of Type VI Secretion system in bacterial genomes. *PLoS ONE* **3**, e2955, doi:10.1371/journal.pone.0002955 (2008).

126 Ren, C. P. *et al.* The ETT2 gene cluster, encoding a second type III secretion system from Escherichia coli, is present in the majority of strains but has undergone widespread mutational attrition. *J. Bacteriol.* **186**, 3547-3560, doi:10.1128/JB.186.11.3547-3560.2004 (2004).

127 Repizo, G. D. *et al.* Differential Role of the T6SS in Acinetobacter baumannii Virulence. *PLoS ONE* **10**, e0138265, doi:10.1371/journal.pone.0138265 (2015).
